# Long-term heating differently impacts diversity and seasonal dynamics of prokaryotes and micro-eukaryotes in Baltic Sea coastal biofilm communities

**DOI:** 10.1093/ismeco/ycag101

**Published:** 2026-04-14

**Authors:** Ida Krogsgaard Svendsen, Iryna Rula, Emelie Nilsson, Johanna Sunde, Songjun Li, Samuel Hylander, Mark Dopson, Anders Forsman, Romana Katerina Salis

**Affiliations:** Centre for Ecology and Evolution in Microbial Model Systems, Department of Biology and Environmental Science, Linnaeus University, Kalmar, SE-39182, Sweden; Centre for Ecology and Evolution in Microbial Model Systems, Department of Biology and Environmental Science, Linnaeus University, Kalmar, SE-39182, Sweden; Centre for Ecology and Evolution in Microbial Model Systems, Department of Biology and Environmental Science, Linnaeus University, Kalmar, SE-39182, Sweden; Centre for Ecology and Evolution in Microbial Model Systems, Department of Biology and Environmental Science, Linnaeus University, Kalmar, SE-39182, Sweden; Centre for Ecology and Evolution in Microbial Model Systems, Department of Biology and Environmental Science, Linnaeus University, Kalmar, SE-39182, Sweden; Centre for Ecology and Evolution in Microbial Model Systems, Department of Biology and Environmental Science, Linnaeus University, Kalmar, SE-39182, Sweden; Centre for Ecology and Evolution in Microbial Model Systems, Department of Biology and Environmental Science, Linnaeus University, Kalmar, SE-39182, Sweden; Centre for Ecology and Evolution in Microbial Model Systems, Department of Biology and Environmental Science, Linnaeus University, Kalmar, SE-39182, Sweden

**Keywords:** biodiversity, biofilm, climate change, community ecology, ecological filtering, functional groups, micro-eukaryote, prokaryote

## Abstract

Warming temperatures, heat waves, and altered conditions associated with climate change affect biodiversity and ecological processes across environments, with coastal zones being particularly vulnerable. Biofilm-forming organisms in shallow coastal areas are taxonomically diverse and include bacteria, fungi, and algae that contribute to energy and nutrient cycling along with providing habitats and food for species at the base of the food web. To understand how biofilm-forming organisms respond differently to spatiotemporally changing environmental conditions, seasonal sampling was performed in a Baltic Sea bay that has undergone 50 years of thermal heating, an unaffected nearby control bay, and a temperature gradient along an exposed coastline between the bays. The diversity, composition, and seasonal dynamics of the biofilm communities differed between the three environments largely due to temperature and water chemistry, with biofilms in the heated bay being more similar across seasons compared with the control bay and the gradient, and with prokaryotes exhibiting stronger spatial heterogeneity and seasonal dynamics compared to micro-eukaryotes. In the gradient, the dominating taxonomic groups were distinct, community composition was primarily influenced by seasonal turnover and wave exposure, and alpha diversity of prokaryotes decreased with increasing temperature. Seasonal shifts in the composition of micro-eukaryotic heterotrophs, phototrophs, and mixotrophs differed between environments, with heterotrophs being more dominant at higher temperatures. In conclusion, these contrasting responses indicated that climate warming may disproportionately impact different components of coastal biofilm communities, potentially decoupling key ecological processes and reducing community resilience in Baltic Sea coastal habitats.

## Introduction

Climate change and associated severe heat waves affect all ecosystems on Earth, threatening biodiversity, food security, and human wellbeing [1–4]. In aquatic environments, climate change brings with it modifications of abiotic conditions such as sea surface levels, wave exposure, oxygen concentrations, salinity, pH, conductivity, and other aspects of water chemistry [5, 6]. In addition, light conditions are altered by reduced ice cover [7] and more frequent and intense algal blooms are expected [8]. Coastal areas are particularly vulnerable to climate change due to faster warming of the water column [9] and play a central role in marine energy and nutrient cycles, providing nearly half the global oceanic primary production [10, 11]. Biofilms in particular are important contributors to ecosystem services in shallow coastal areas, as they harbor primary producers, contribute to nutrient cycling, and provide habitats and food sources for a variety of microorganisms and small invertebrates, forming the base of the food web [12–14].

Higher temperatures in coastal benthic waters reduce bacterial diversity and modify the composition and activity of biofilms [15, 16]. Together with species interactions [17], disturbances, nutrient levels, and water chemistry also affect the composition and dynamics of biofilms [18], contributing to vertical and horizontal spatial structure and temporal shifts [19, 20]. Conversely, modifications of biofilm composition may disrupt broader microbial diversity and impose cascading effects by affecting biogeochemistry and impacting organisms at other trophic levels that rely on biofilms for food or habitat [13, 21, 22].

The ecology of biofilms has attracted considerable scientific attention. According to a topic literature search on ISI Web of Science, >17 000 biofilm papers were published by June 2025, but the existing literature is characterized by taxonomic and environmental biases (Fig. S1). For example, biofilms comprise both prokaryotic and micro-eukaryotic taxa, and these differ in complexity of cellular structures, generation time, ecological function, and in their ability to cope with and recover from environmental changes [23–25]. Despite this, only a minority of studies have combined the use of 16S and 18S ribosomal Ribonucleic acid (rRNA) gene molecular markers that target these different groups (Fig. S1), limiting the ability to compare their responsiveness to environmental stressors. Less than 4% of existing studies used both marker types to examine biofilms in brackish and marine ecosystems (Fig. S1), despite that these in terms of volume make up ~99.9% of surface water bodies globally [26].

To better understand how climate change impacts biodiversity and the relative abundance of different trophic groups requires long-term experiments conducted at spatiotemporal scales relevant for the species and community under investigation, and complex conditions that capture the combined additive and interactive effects of temperature and other environmental factors [27]. Specifically, biofilms comprise three main trophic groups: phototrophs, heterotrophs, and mixotrophs. As temperatures increase, the metabolic rates increase across trophic groups, but heterotrophs are generally more temperature-responsive than phototrophs, often leading to a shift towards their dominance [28, 29]. Mixotrophs are capable of switching between heterotrophy and phototrophy and thus exhibit greater flexibility in responding to varying environmental conditions [30] and their relative abundance may therefore be more stable. To address these important issues and impacts of climate change, this study investigated the spatiotemporal dynamics of biofilms in three distinct thermal environments in a coastal area of the Baltic Sea.

The Oskarshamn Nuclear Power Plant (OKG) in southeastern Sweden uses Baltic Sea water for cooling and has discharged heated water into a nearby bay for >50 years (Fig. S2). This has raised the water temperature by an average of 5.1°C compared to a nearby (distance ~1.5 km) control bay, with the temperature difference between bays being greatest during winter [31]. This temperature increase is within the predicted range for the RCP5-8.5 scenario by the year 2100 of 3.3°C–5.7°C [32, 33], which may occur in coastal areas under less extreme predictions [20]. This system thus provides a unique opportunity to study the effects of long-term warming and future climate conditions in a large-scale Baltic Sea coastal area under the influence of seasonally fluctuating conditions and complex multi-species interactions in a close to natural setting.

Previous comparisons in this system show that heating has altered the diversity, structure, seasonal dynamics, and activity of microbial communities in the water column and bottom sediments, as well as resulted in elevated RNA transcript counts assigned to stress related genes, fewer cells potentially due to a portion of the metabolic energy being diverted to heat-related stress response rather than cell division, modified biogeochemical processes, and reshaped greenhouse gas fluxes in the sediments [20, 31, 34–40]. However, earlier studies have focused on prokaryotic communities (but see [38]), in bottom water and sediment samples collected at relatively deep waters (mean across studies = 5.6 m, range = 1.2–30 m).

The present study investigated spatiotemporal variation in the diversity and composition of prokaryotic and micro-eukaryotic sessile biofilm communities on hard substrates (stones) in near shore benthic habitats characterized by pronounced environmental fluctuations in the abovementioned heated bay, the nearby control bay, and in the gradient environment along a more wave exposed coastline between the bays that is characterized by a decrease in temperature from the heated bay towards the entrance of the control bay (Fig. S2). It was hypothesized that: (i) the diversity, composition, and seasonal dynamics of the biofilm communities will differ between the three environments; (ii) the micro-eukaryotes will show less differences between bays and exhibit weaker seasonal dynamics, compared with the prokaryotes; (iii) the biofilm communities in the gradient will vary in accordance with the temperature and wave exposure, particularly for prokaryotes; and that (iv) the composition of different trophic groups will differ both between environments and over time, with heterotrophs being relatively more dominant at higher temperatures and mixotrophs showing less temporal fluctuations.

## Materials and methods

### Study area, sampling sites, and sampling methods

Samples of biofilm communities were collected from natural stones in shallow coastal waters (<1 m) at 8–10 sites per environment (the heated bay, nearby control bay, and the temperature gradient between the bays, Fig. S2; Supplementary Materials and Methods) on six occasions between June 2021 and October 2022 (coordinates and depths in Table S1). During the first sampling (June 2021), biofilm was collected at two depths (43 ± 12 cm and 78 ± 14 cm) at each site to compare within-site depth variation, while subsequent samplings only included the shallower depth. In total, 173 stone biofilm samples were obtained from stones (1 stone per site/depth/environment/time; Table S2). Additional biofilm samples were collected from HOBO-loggers deployed at deeper depths (4 ± 1.4 m) in the heated and control bays (eight and seven sites, respectively; Table S3) in August 2021 and recovered in September 2022 [41]. Field sampling of biofilm was done using sterile tools, with 20 cm^2^ area collected from stones and the full surface area from HOBO-loggers. Samples were then frozen until processing. Further details of the study area and sampling are provided in the Materials and Methods section of the supplementary information.

### Sample processing and analysis

Biofilm samples were homogenized and DNA extracted using the ZymoBIOMICS DNA Miniprep Kit (BioSite Nordic). Prokaryotic 16S rRNA genes (V3–V4) were amplified with 341F + 805R primers [42,43] and micro-eukaryotic 18S rRNA genes (V4) with TAReuk454FWD1 + TAReukREV3_modified primers [44,45]. Amplicons were sequenced on Illumina MiSeq (16S) and NextSeq (18S) platforms at SciLifeLab, Sweden. *In situ* environmental parameters (temperature, oxygen, conductivity) were measured at each site on each sampling occasion (for both stones and HOBO-loggers) and water chemistry samples (Si, P, N) were collected monthly from the stone sampling sites. A more detailed description of the sampling protocols and analyses can be found in the Material and Methods section of the supplemental information.

### Sequencing and statistical analysis

Raw reads were processed using nf-core ampliseq (v.2.9.0) [46], implementing DADA2 (v.1.30.0 or v.1.22.0) [47] to infer amplicon sequencing variants (ASVs) and assign taxonomy. The SBDI-GTDB database (Release R07-RS207-1) was used for classification of 16S, and the PR^2^ database v.5.0.0 [48] for 18S rRNA genes sequences. Prokaryotic ASVs assigned to chloroplasts or mitochondria were removed. For micro-eukaryotes, only Eukaryota were retained, excluding Metazoa and Embryophyta. Remaining ASVs were further classified as phototrophs, heterotrophs, or mixotrophs with the Mixoplankton Database (MDB) [49] being used to identify mixotrophic species. For ASVs belonging to species not found in the MDB, trophic groups were assigned according to taxonomic classification at supergroup, division, subdivision, and class level (see Table S4). After quality filtering, 16S rRNA gene samples contained 42 399–241 781 reads per sample (mean ± SD: 83574 ± 25 629) while 18S rRNA gene samples contained 28 548–1 400 786 reads per sample (422 331 ± 232 555). One 18S sample with only 3077 reads was excluded. Rarefaction curves (Fig. S3) indicated adequate sampling depth. For alpha diversity analyses, data were rarefied to the lowest sequencing depth within each dataset using *MicrobiomeStat*.

All analyses and figures were produced in R (v.4.3.1) [50]. Linear mixed-effects models were used to assess the effects of environment, sampling date/season, and/or water depth on alpha diversity (Shannon index), unique ASVs, and the relative abundance of eukaryotic trophic groups. Site was included as a random factor, significance evaluated using Type III Analysis of Variance with Satterthwaite’s method (*lme4*, *lmerTest* packages), and pairwise comparisons with Tukey adjustment (*emmeans*). Beta diversity was evaluated using Aitchison distances, visualized by principal coordinates analysis (PCoA), and tested with permutational multivariate analysis of variance (perMANOVA; 9999 permutations, *vegan*). Euler diagrams were used to quantify shared and unique ASVs among environments (*MicEco*). Relationships between community composition and environmental variables were examined with redundancy analysis (RDA) on rCLR-transformed data (*vegan*). A more detailed description is provided in the supplemental information (Material and methods, Table S5).

## Results

### Spatial variation in biofilm communities during summer in relation to environment, sampling site, and water depth

Biofilm rRNA gene-based results for samples collected on the first sampling occasion (June 2021) were initially analyzed separately, since these data contained stones collected at two different depths (~43 cm versus 78 cm). For the alpha diversity of prokaryotes, there was a significant effect of the interaction between water depth and environment (*F_2,105_* = 3.22, *P* = .043; Table S6, Fig. S4a). The interaction reflected that diversity was highest overall in the control bay, increased with increasing water depth in both the control and the heated bays, and was independent of water depth in the gradient. For micro-eukaryotes there were only significant main effects of the environment (*F_2,75_* = 5.46, *P* = .006) and water depth (*F_2,107_* = 9.49, *P* = .002), with diversity being highest in the heated bay and increasing with water depth (Table S6, Fig. S4b).

**Figure 1:**
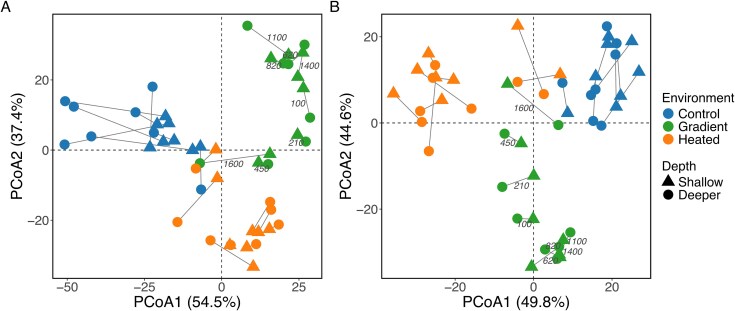
PCoA plot based on Aitchison distance, showing the community composition of prokaryotes (A) and micro-eukaryotes (B) in the heated bay, control bay, and gradient. The figure is based on data collected in June 2021. Each point represents a sample from one site. Colors indicate environment while shapes represent water depth (one shallower, 43 ± 12 cm; and one slightly deeper, 78 ± 14 cm, Table S1). The axes represent the first two principal coordinates, which explain the majority of the variation in the data. Samples in the gradient are annotated with the distance (m) from the heated bay.

The rRNA gene ASVs based community composition differed between environments for prokaryotes (perMANOVA: *F_2,42_* = 45.47, *P* = .001, *R^2^* = 0.66) but not for micro-eukaryotes (*F_2,42_* = 47.91, *P* = .001, *R^2^* = 0.69), and was independent of water depth for both groups (Table S7, Fig. 1). This indicated that water depth within the sampling range had limited biological influence compared to the environment.

In general, the rRNA gene-based community composition of samples collected from different sites within each of the three environments clustered closer to each other than samples from different environments (Fig. 1). There was also a greater similarity in biofilm community composition between stones at the two different water depths within a sampling site than between different sampling sites (Fig. 1). The perMANOVA results showed that site identity explained a significant proportion of variance both across all environments (16S: *R*^2^ = 0.23, *P* < .001; 18S: *R*^2^ = 0.24, *P* < .001) and within individual environments. For the gradient, where differences in wave exposure and turbulence increased patchiness, site explained 84 and 89% of variance for prokaryotic and micro-eukaryotic communities, respectively (16S: *R*^2^ = 0.84, *P* < .001; 18S: *R*^2^ = 0.89, *P* < .001). Similar patterns were observed for the heated bay (16S: *R*^2^ = 0.79, *P* = .003; 18S: *R*^2^ = 0.75, *P* = .011). In the control bay, site identity explained a moderate albeit non-significant proportion of variance (16S: *R*^2^ = 0.45, *P* = .49; 18S: *R*^2^ = 0.54, *P* = .26). These results demonstrated that site-level differences were substantially larger than within-site variation (including depth-related and micro-scale patchiness), even in the gradient environment. Sampling only from the shallower depth at each sampling site on each sampling occasion for the remainder of the study was therefore deemed sufficient to reliably characterize spatiotemporal variation in biofilm communities.

Regarding drivers of variation in community composition of prokaryotes, the RDA showed significant effects of temperature, conductivity, and water depth (Table 1, Fig. S5a). The composition of micro-eukaryotes was significantly influenced by temperature and conductivity, but not by water depth or oxygen (Table 1, Fig. S5b). For information on the most dominant taxonomic taxa (classes) see supplementary information (Results, Fig. S6).

**Table 1:** Statistics for the RDAs showing associations of biofilm community composition with environmental factors (water temperature, water chemistry, and water depth).

RDA		Prokaryotes				Micro-eukaryotes			
Experiment	Parameter	NUMDF; DENDF	Variance (%)	*F*	*P*	NUMDF; DENDF	Variance (%)	*F*	*P*
June 2021	Temperature	1; 43	20.93	14.65	**.001**	1; 43	27.79	20.43	**.001**
June 2021	Conductivity	1; 43	9.74	6.82	**.001**	1; 43	11.36	8.35	**.001**
June 2021	Oxygen	1; 43	2.29	1.60	.208	1; 43	2.27	1.67	.174
June 2021	Depth	1; 43	5.62	3.94	**.014**	1; 43	0.10	0.07	.966
Shallow stones	Temperature	1; 93	13.96	16.94	**.001**	1; 92	19.32	26.3	**.001**
Shallow stones	Conductivity	1; 93	1.12	1.36	.272	1; 92	4.19	5.7	**.002**
Shallow stones	Oxygen	1; 93	0.54	0.65	.583	1; 92	0.05	0.07	.972
Shallow stones	Silicon	1; 93	4.52	5.48	**.003**	1; 92	4.91	6.68	**.002**
Shallow stones	Nitrogen	1; 93	1.69	2.05	.114	1; 92	3.53	4.81	**.004**
Shallow stones	Phosphorus	1; 93	1.50	1.82	.143	1; 92	0.39	0.53	.629
HOBO-loggers	Temperature	1; 10	40.94	10.36	**.001**	1; 10	32.17	8.13	**.002**
HOBO-loggers	Conductivity	1; 10	1.50	0.38	.734	1; 10	3.37	0.85	.455
HOBO-loggers	Oxygen	1; 10	1.96	0.5	.647	1; 10	6.21	1.57	.199
HOBO-loggers	Depth	1; 10	16.11	4.08	**.023**	1; 10	18.68	4.72	**.016**

The results are based on separate analyses of prokaryotes and micro-eukaryotes, using data from three different sample collections (“experiments”) that differ with regards to spatial and temporal heterogeneity. Values in bold indicate statistical significance, *P* < .05.

### Contrasting seasonal variation of biofilms in the three environments

**Figure 2:**
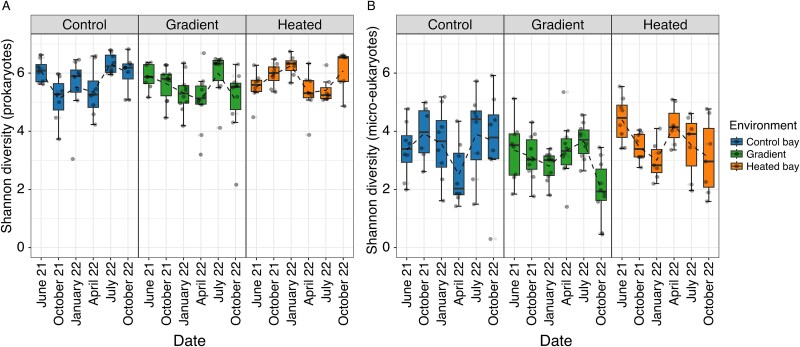
Spatial and seasonal variation in rRNA gene-based alpha diversity of prokaryotes (A) and micro-eukaryotes (B). Data for biofilm samples collected from shallower depths at the sites in three environments (control bay, gradient , heated bay) on six occasions. Boxplots show median and interquartile range with individual data points overlaid (*n* = 7–10 per bay per timepoint, see Table S2 for details). Results for samples collected during different seasons: summer (June 2021, July 2022), autumn (October 2021, October 2022), winter (January 2022), and spring (April 2022).

**Figure 3:**
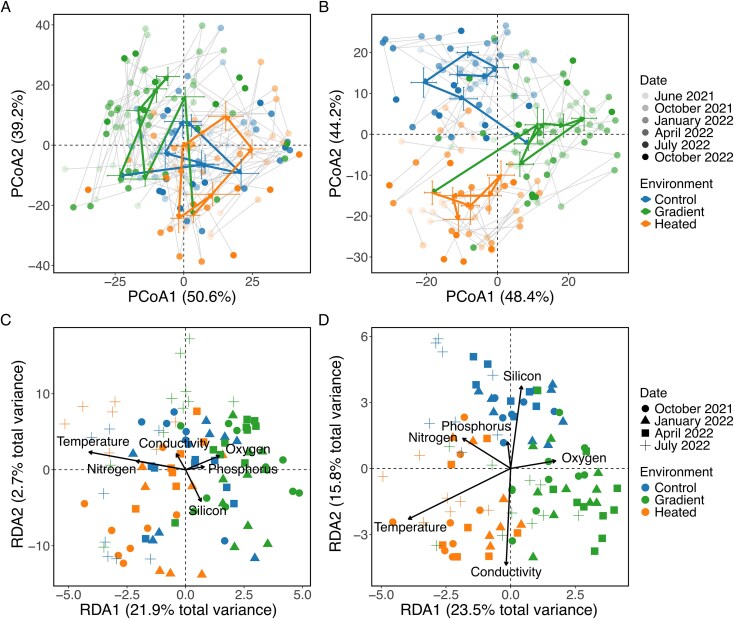
Variation and change in prokaryotic and micro-eukaryotic biofilm community composition according to location, season, and abiotic conditions. The top panels show Aitchison distance based PCoA plots visualizing the spatiotemporal variation in biofilm rRNA gene-based community composition among samples according to environment and sampling occasion for prokaryotes (A) and micro-eukaryotes (B). The arrows indicate the directions and magnitudes of the average temporal changes between sampling occasions. The bottom RDA plots visualize the association of community composition with temperature and water chemistry for prokaryotes (C) and micro-eukaryotes (D). The arrows indicate the direction and strength (length) of the relationships, and the percentages of constrained variance are displayed. For all panels, the points represent individual samples, with colors indicating environment and shapes indicating sampling occasion. Results for samples collected during different seasons: summer (June 2021, July 2022), autumn (October 2021, October 2022), winter (January 2022), and spring (April 2022).

The data from all six sampling occasions showed that the seasonal patterns of variation in ASV-level alpha diversity (Fig. 2) and community composition (Fig. 3) of both prokaryotes and micro-eukaryotes, as well as the relative abundance of ASVs representing different trophic groups in the biofilm communities, differed between the three environments and with functional stability being greatest in the heated bay.

The alpha diversity of prokaryotes was higher overall in all three environments than that of micro-eukaryotes (mean Shannon diversity index ~5.5 vs. ~3.5; Fig. 2). The difference in seasonality between environments was significant for prokaryotes (sampling occasion × environment, *F_10,131_* = 2.85, *P* = .003) and micro-eukaryotes (*F_10,109_* = 2.77, *P* = .004; Fig. 2), reflecting that temporal fluctuations in alpha diversity were not synchronous across environments. There was a significant main effect of sampling occasion on alpha diversity for both groups, but the main effect of environment was only significant for micro-eukaryotes (Table S6). When pooling data across all three environments, prokaryote alpha diversity increased significantly with increasing water temperature (LMM: *F_1,164_* = 7.66, *P* = .006; estimate = +0.04 ± 0.015 per °C), whereas micro-eukaryote alpha diversity decreased (*F_1,165_* = 6.80, *P* = .010; estimate = −0.06 ± 0.023 per °C).

Regarding community composition, the separation among environments changed over time for prokaryotes (perMANOVA, sampling occasion × environment: *F_10,131_* = 3.41, *P* = .001, *R^2^* = 0.13) and micro-eukaryotes showed a similar albeit non-significant tendency (*F_10,130_* = 1.51, *P* = .061, *R^2^* = 0.04) (Table S7, Fig. 3). Overall, community composition was more similar within than between environments, and samples from the temperature gradient did not cluster distinctly between the heated and the control bay. Community composition was also more similar within than between seasons, with temporal shifts being most pronounced in prokaryotes (Fig. 3).

Water temperature and most of the measured water chemistry variables differed between the three environments and changed over time (Fig. S7). The RDAs further demonstrated that the spatial variation and temporal shifts in community composition of both prokaryotes and micro-eukaryotes were influenced most strongly by temperature, followed by silicon, conductivity, and nitrogen (in micro-eukaryotes), but not by oxygen concentration or phosphorus (Table 1, Fig. 3c and d).

The domination of taxonomic classes was not uniformly distributed across the sampling sites, environments, and sampling occasions. For details see supplementary information (Results, Figs S8 and S9).

### Influences of alpha diversity and community composition of biofilms in the gradient

In the gradient, prokaryotic alpha diversity decreased overall with increasing water temperature (Table S8, Fig. S10a). In contrast, the micro-eukaryotic alpha diversity was independent of temperature (Table S8**,**  Fig. S10b). Sampling occasion was significant for both prokaryotes and micro-eukaryotes, while wave exposure had no significant effect on alpha diversity in either group (Table S8).

Regarding community composition, temperature had a weak but significant effect on micro-eukaryotes (*R^2^* = 0.05) and a marginally non-significant effect on prokaryotes (*R^2^* = 0.02). Seasonal turnover was the dominant driver of community composition in both prokaryotes (*R^2^* = 0.31) and micro-eukaryotes (*R^2^* = 0.28, Table S8). Wave exposure also strongly influenced the composition of both groups, explaining a substantially larger proportion of variance in micro-eukaryotes (*R^2^* = 0.24) than in prokaryotes (*R^2^* = 0.11), and compared with the influence of dissolved oxygen and phosphorus (Table S8).

### Seasonality of unique and shared amplicon sequencing variants

**Figure 4:**
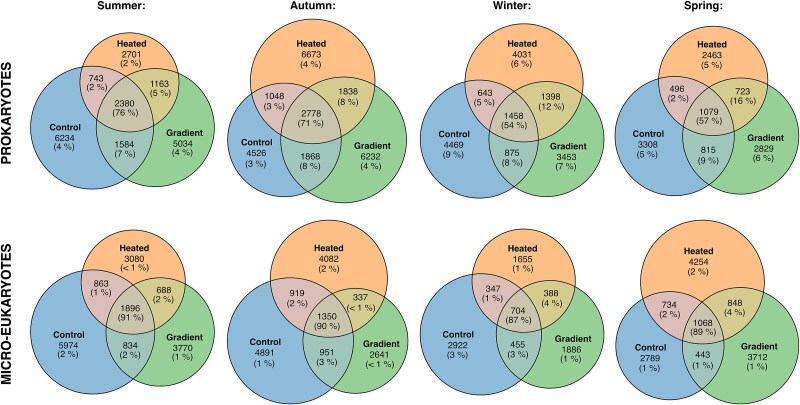
Euler diagrams illustrating the number of unique and shared rRNA gene ASVs for prokaryotes (top panel) and micro-eukaryotes (bottom panel) in biofilm samples collected in different seasons from three environments: heated bay, control bay, and temperature gradient between them. The Euler diagrams include all ASVs present in each environment. The number of unique and shared ASVs are displayed and the sizes of the circles are proportional to the number of ASVs. The relative abundance (% of total sequences) of the unique and shared ASVs is shown in parentheses. Results for samples collected during summer (June 2021, July 2022), autumn (October 2021, October 2022), winter (January 2022), and spring (April 2022).

The relative abundance (% of total sequences) of the rRNA gene ASVs that were shared between all three environments was lower for prokaryotes than for micro-eukaryotes (~60% vs. ~90%), particularly during winter and spring (Fig. 4). Conversely, there were more ASVs that were unique to a given environment among the prokaryotes than among the micro-eukaryotes (~5% vs. ~2%). For prokaryotes (but not micro-eukaryotes), more ASVs in the heated bay were shared with the gradient than with the control bay, in all seasons (Fig. 4).

**Figure 5:**
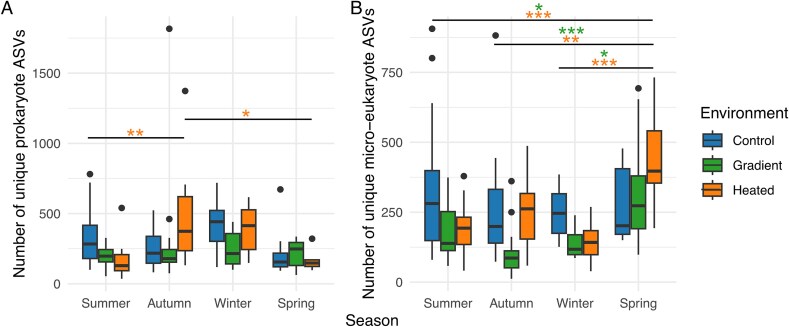
Seasonal dynamics of unique rRNA gene-based ASVs for prokaryotes (A) and micro-eukaryotes (B) across different environments: control bay, temperature gradient, and heated bay. Box plots show the number of unique ASVs for each environment during summer (June 2021, July 2022), autumn (October 2021, October 2022), winter (January 2022), and spring (April 2022). Each data point represents one sample and unique ASVs were defined as those present in at least one sample from a given environment but absent from all samples in the other two environments. Asterisks indicate significant differences between seasons based on pairwise comparisons tests (^*^P < .05, ^**^P < .01, ^***^P < .001).

More in-depth analyses based on the number of unique ASVs per site showed that for prokaryotes there was a significant interaction between environment and season (*F_6,116_* = 2.24, *P* = .044; Fig. 5a). Pairwise comparisons suggested that the number of unique ASVs did not vary across seasons in the temperature gradient, while the strongest seasonal differences were observed in the heated bay (Fig. 5a). In autumn, the number of unique ASVs was highest in the heated bay, whereas the number of unique ASVs in the heated bay decreased in spring (April) and summer (June, July).

For micro-eukaryotes there was also a significant interaction between environment and season (*F_6,114_* = 3.02, *P* = .009; Fig. 5b). The number of unique micro-eukaryotic ASVs did not vary across seasons in the control bay. However, in the heated bay and the gradient the number of unique ASVs was higher in spring (April).

**Figure 6:**
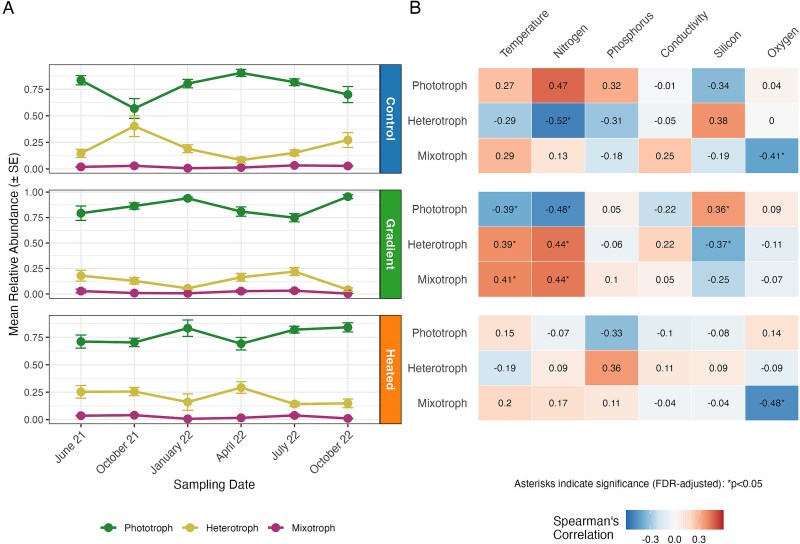
Temporal variation and environmental correlations of micro-eukaryotic trophic groups in biofilm communities in the three environments. (A) Mean (± SE) relative abundance of rRNA gene-based ASVs representing phototrophs, heterotrophs, and mixotrophs across six sampling occasions in the three environments (from top to bottom, control bay, temperature gradient, and heated bay). (B) Heat maps of Spearman correlation coefficients showing associations between the relative abundances of the three trophic groups and water temperature and the water chemistry variables in each environment (from top to bottom, control bay, temperature gradient, and heated bay). Color gradients range from dark blue (strong negative correlation) to dark red (strong positive correlation). Asterisks indicate statistically significant correlations after FDR adjustment (*P* < .05).

With regards to micro-eukaryote trophic groups, the results indicated that seasonal shifts in their relative abundance were not synchronous in the three environments (environment × date, phototrophs: *F_10,108_* = 4.04, *P* < .001; heterotrophs: *F_10,130_* = 4.13, *P* < .001; Fig. 6a). Phototroph relative abundance was lowest in autumn (October 2021 and 2022) in the control bay, and highest in winter (January) and autumn (October 2022) in the gradient, whereas heterotrophs showed the opposite pattern. Neither phototrophs nor heterotrophs showed significant seasonal variation in the heated bay. In contrast, the relative abundance of mixotrophs, which were relatively much less abundant than phototrophs and heterotrophs, was independent of environment but differed significantly with sampling date (*F_5,109_* = 5.27, *P* < .001), being lowest in winter (January) and highest in summer (July) (Fig. 6a).

The drivers of trophic group variation differed among the three environments (Fig. 6b). In the gradient, spatiotemporal variation in relative abundances of the three trophic groups was most strongly associated with temperature and nitrogen concentrations, with contrasting effects on phototrophs (negative correlations) versus heterotrophs and mixotrophs (positive correlations). In the control bay, nitrogen was the dominant correlate, showing a strong negative association with heterotrophs and a positive but non-significant association with phototrophs (Fig. 6b). In the heated bay, none of the measured environmental variables were significantly associated with phototroph or heterotroph relative abundance. Oxygen concentration was negatively associated with mixotroph relative abundance in the control and heated bays, but not in the gradient.

### Biofilm communities in deeper waters differed between the heated and control bay

The alpha diversity of prokaryotes and micro-eukaryotes in biofilm samples collected from HOBO-loggers submerged for one year in deeper (4 ± 1.4 m) waters in the heated and control bay was not significantly influenced by the environment, water depth, or their interaction (all *P* > .05; Table S6, Fig. S11). However, the perMANOVA results showed that community composition of prokaryotes varied significantly according to environment (*R^2^* = 0.47) and the water depth of the HOBO-loggers (*R^2^* = 0.26) but was not affected by their interaction (Table S7). The community composition of micro-eukaryotes varied significantly only between the two bays (*R^2^* = 0.51, Table S7).

**Figure 7:**
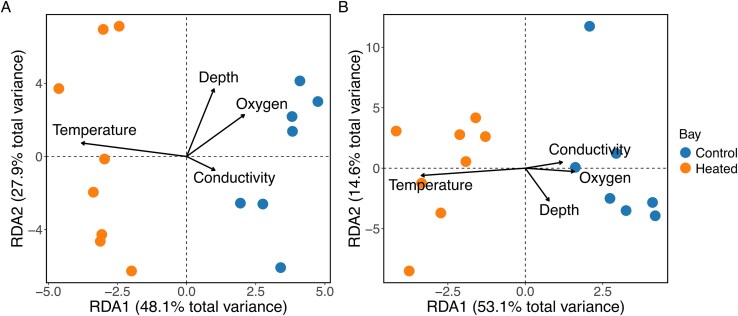
RDA plot illustrating the distribution of rRNA gene-based prokaryotes (A) and micro-eukaryotes (B) in biofilm communities across different sample sites in the heated and the control bay according to environmental factors. Arrows indicate the direction and strength of the relationships between environmental factors (water temperature, water depth, oxygen concentration, and conductivity) and biofilm communities. Due to insufficient amplification of samples, data from the lower HOBO-loggers were excluded and only data from the upper HOBO-loggers were used for this analysis.

The RDA results suggested that the composition of both prokaryotes and micro-eukaryotes depended on water temperature and water depth (Fig. 7, Table 1). There was no significant effect of conductivity or oxygen concentration. For information on the dominating prokaryotic and micro-eukaryotic taxonomic classes at deeper waters in the heated and control bay see supplementary information (Results, Fig. S12).

## Discussion

Overall, this study supported the first hypothesis that the diversity, composition, and seasonal dynamics of the biofilm communities on hard benthic substrates in near-shore shallow habitats differed across the three investigated environments. The initial summer sampling showed that although there were substantial small-scale spatial differences among sites within each environment, community composition generally varied less between the different water depths within sampling sites than among sites within environments, and that there were distinct differences between the three environments.

### Spatiotemporal patterns

The temporal sampling revealed contrasting and non-synchronous seasonal shifts in both alpha diversity and composition of the biofilm communities in the two bays and the gradient, particularly in prokaryotes. This likely reflects the direct and indirect effects that temperature has on biological processes [51–54] and on the chemical and physical properties of water [55,56] in combination with divergent ecological filtering and selection mediated by distinct abiotic conditions and species interactions in the different environments [57–59]. Although temperature and water chemistry (silicon, conductivity, phosphorus, nitrogen, and oxygen) were identified as driving factors in this and previous studies [60–63], it is important to acknowledge that these measured variables explain only a portion of the observed community variation. Our analyses showed that environment and sampling occasion consistently accounted for more variance than individual measured physicochemical parameters, indicating that spatial and temporal variation in other factors may also have contributed to the observed patterns. Such potential factors included but were not limited to biotic interactions (e.g. grazing by benthic invertebrates, microbial competition and facilitation), stochastic processes, microhabitat heterogeneity, and other environmental variables not measured in this study, such as light conditions, water turbulence, bottom substrate characteristics, ice cover, and vegetation cover (mainly macroalgae) [7,62,64,65].

This study’s results were qualitatively similar to those in previous investigations conducted in the same area, demonstrating that ~50 years of heating has led to alterations in the diversity, structure, and composition of the microbial communities in bottom waters and sediments [31, 35–37]. Thus, this present study adds a layer of generality by extending these patterns to prokaryotic and micro-eukaryotic taxa in near-shore shallow biofilm communities on hard substrates. Zooming out, the overall findings were in agreement with the results of studies conducted in other areas, reporting effects of temperature on diversity, composition, and dynamics of biofilms in both freshwater and marine environments [53,66–68].

The diversity, composition, and relative abundance of phototrophs and heterotrophs of biofilm communities fluctuated less in the heated bay with higher and relatively more stable temperatures, compared with the control bay and the temperature gradient. This is indicative of higher functional stability, and consistent with recent experimental evidence that fixed warming enhances Shannon diversity, compared to fluctuating temperatures [53]. Similar results were previously reported for microbial communities in surface sediments (0–1 cm) in this same model system [35]. It is also noteworthy within the context of extreme environments and marine heat waves [69] that the number of unique prokaryote and micro-eukaryote ASVs varied most strongly among seasons in the heated bay.

### Dominating taxa in shallow and deeper water

Ecological and environmental conditions become increasingly stable and homogeneous with greater water depth [70,71]. This may partly explain why the biofilm communities collected from the HOBO-loggers that had been submerged for one year in the heated and control bays did not differ in alpha diversity, and why the community composition of both prokaryotes and micro-eukaryotes differed between bays according to water temperature while being independent of water chemistry.

It has been established that biofilm communities differ according to substrate type [14,18,72]. Together with the fact that the biofilms on the plastic HOBO-loggers had developed over the course of a year in relatively deep waters, whereas the samples from stones in shallow sites represented older biofilms, this prevented discriminating between substrate and water depth effects. However, it was noticeable that among prokaryotes, the three most dominant taxa found in the deeper water biofilms on HOBO-loggers (Cyanobacteria, Alphaproteobacteria, and Gammaproteobacteria) were also among the most dominant taxa in biofilms on the shallow stones. For the micro-eukaryotic taxa, the most dominant taxa in the deeper water HOBO-loggers (Bacillariophyceae, Florideophyceae, Phaeophyceae, and Ulvophyceae) were also dominant on the shallower stones.

### Differences between prokaryotes and micro-eukaryotes

In agreement with the second hypothesis, micro-eukaryotes showed fewer differences between the two bays, and exhibited weaker seasonal dynamics, compared with prokaryotes. That prokaryotes in the biofilm varied more than micro-eukaryotes between environments and across seasons in both alpha diversity and community composition was consistent with previous demonstrations that these taxa exhibit distinct variations in cellular structure complexity, generation time, mutation rates, specific ecological functions, niche breadth, and in the capacity to endure and recover from environmental changes [23–25]. The different thermal and ecological conditions in the three environments likely favored different taxa in both prokaryotes and micro-eukaryotes, resulting in local environmental selection [73,74]. Some of the differences seen in the heated bay may also reflect differential thermal acclimation of organisms in that environment [53].

The results for alpha diversity were mirrored overall by the analysis of unique and shared rRNA gene ASVs, both indicating more pronounced differences between environments in prokaryotes. The relative abundance of ASVs that were shared across all three environments was lower (~60%) compared to micro-eukaryotes (~90%) and conversely, more prokaryotic than micro-eukaryotic ASVs were unique to each environment. However, micro-eukaryotes exhibited stronger seasonal differences in the number of unique taxa compared to prokaryotes. That the two groups responded differently to environmental conditions was further supported by the fact that their seasonal shifts were not synchronous and manifested most strongly in different environments, potentially reflecting overall differences in generation time, adaptive potential, and niche selection [25].

### The temperature gradient

In agreement with the third hypothesis, biofilm communities in the gradient partially varied in accordance with water temperature and wave exposure, particularly in prokaryotes, with alpha diversity generally decreasing with increasing temperature. This is consistent with suggestions that higher temperatures can decrease prokaryotic alpha diversity due to thermal stress, faster resource depletion, and increased competition [15,16,66,67,75].

Besides variable temperature conditions, the gradient is characterized by considerable variation in wave exposure (ranging from extremely sheltered to moderately exposed) and turbulence. Physical instability imposes stress [76] and may induce shifts towards fast-growing, stress-tolerant taxa [53,74,77]. The compositional patterns identified by the results strengthened the conclusion that the distinct, unstable, and spatially heterogeneous environmental conditions in the temperature gradient impacted the structure of biofilm communities. For example, the June 2021 sampling indicated that differences among sites accounted for ~85% of the variance in community composition of both prokaryotes and micro-eukaryotes. The analyses also identified seasonal turnover and wave exposure as dominant drivers of community composition in the gradient. Notably, more prokaryote ASVs in the heated bay were shared with the gradient than with the control bay. This pointed to an effect of directional colonization mediated by the currents created by the discharge of cooling water from the reactor into the heated bay and spreading further into the southern part of the gradient.

### Trophic groups

In partial agreement with the fourth—and final—hypothesis, the composition of micro-eukaryotic heterotrophs, phototrophs, and mixotrophs differed both between environments and over time, and with heterotrophs being relatively more dominant at higher temperatures. This conforms with previous findings that factors such as temperature [15], light availability [65], nutrient levels [78], and water chemistry can significantly influence the distribution and abundance of micro-eukaryotic trophic groups [63]. That micro-eukaryotic heterotrophs are favored by warmer conditions is in agreement with previous studies [79,80], potentially reflecting increased organic matter availability [81].

While functional flexibility may allow mixotrophs to better cope with fluctuating environmental conditions, the present findings do not support a more stable relative abundance of mixotrophs in the gradient. Instead, their relative abundance varied non-synchronously across environments, with seasonal fluctuations being most pronounced in the gradient. However, conclusions regarding mixotrophs should be interpreted with some caution as their relative abundance may be underestimated due to identification challenges [82] and incomplete reference databases [83]. Additionally, our reliance on broad taxonomic classifications (e.g. including all Dinoflagellata) based on RNA gene sequencing cannot capture the metabolic flexibility of many micro-eukaryotes, whose nutritional strategies can shift with environmental conditions [84–86]. Further physiological characterization, metatranscriptomics, or stable isotope probing approaches are needed to provide more detailed insights into active metabolism and nutritional strategies.

## Conclusions

This study demonstrated that spatiotemporal variation in environmental conditions and prolonged thermal heating (comparable to the expected temperature increase for the Baltic Sea by 2100) differently impacted the alpha diversity, community composition, and seasonal dynamics of prokaryotic versus micro-eukaryotic taxa in biofilms inhabiting hard benthic substrates in shallow near-shore Baltic Sea coastal habitats. Other studies in the same system suggest comparable effects of warming on prokaryotic microbial communities in sediments, bottom water, and surface water, as well as elevated stress levels and reduced microbial abundance [20, 31, 34–37, 40]. The novel results for sessile biofilms, the demonstration of contrasting responses of prokaryotes and micro-eukaryotes, and the documented spatiotemporal dynamics of different trophic groups with heterotrophs dominating under warmer conditions, together with the inclusion of the temperature gradient environment in the present study add generality and broadened knowledge that can help predict the influence of future global warming on the structure and functioning of coastal ecosystems. Future work, such as repeated experimental introductions of novel substrates and reciprocal translocations between environments at different seasons, can inform about the mechanistic underpinnings and further our understanding of how biotic interactions and changing environmental conditions impact the assembly, dynamics, productivity, functional properties, resilience, and reversibility of biofilm communities.

## Supplementary Material

Svendsen_et_al_Supp_Info_20260408_ycag101

Table_S5_ycag101

## Data Availability

The raw sequencing data have been uploaded to the NCBI Sequence Read Archive (SRA) under BioProject accession number PRJNA1338476. All raw data used for the analyses are available in the Zenodo repository: https://doi.org/10.5281/zenodo.17241761.

## Code availability

All the R code used for the analyses are available in the Zenodo repository: https://doi.org/10.5281/zenodo.17241761.
