# Supplementary material for: Long-term heating differently impacts diversity and seasonal dynamics of prokaryotes and micro-eukaryotes in Baltic Sea coastal biofilm communities
Source: ISME Commun. 2026 Apr 14;6(1):ycag101. doi: 10.1093/ismeco/ycag101 (PMC13174331; doi:10.1093/ismeco/ycag101)
Supplement: Svendsen_et_al_Supp_Info_20260408_ycag101 [file svendsen_et_al_supp_info_20260408_ycag101.pdf]

Supplementary Information for

Long-term heating differently impacts diversity and seasonal dynamics of prokaryotes and micro-eukaryotes in Baltic Sea coastal biofilm communities

Ida Krogsgaard Svendsen, Iryna Rula, Emelie Nilsson, Johanna Sunde, Songjun Li, Samuel Hylander, Mark Dopson, Anders Forsman[†] & Romana Katerina Salis[†*]

[†] These authors contributed equally, should be considered joint senior authors and have the right to place their name last in the CV.

Romana Salis
E-mail: romana.salis@lnu.se

This PDF file includes:

Supplementary Materials and Methods
Supplementary Results
Tables S1-S8
Figures S1-S12
Supplementary References

Supplementary Material and Methods

Study area

Samples of near-shore biofilm communities were collected on six occasions in three different environments: a Baltic Sea bay that has been heated for over 50 years, a nearby control bay unaffected by heating, and along the temperature gradient coastline between the heated and the control bays, on the east coast of Sweden (**Fig. S2**). The gradient was a more open habitat, compared with the two bays, with more wave exposure and turbulence known to disrupt biofilm communities [1] while also enhancing nutrient distribution [2], which can support a more diverse biofilm [3]. Additionally, biofilm communities were collected on plastic temperature loggers incubated in deeper waters (~4 m) for one year in the heated and control bays, respectively (see below). The heated and control bays were wind-sheltered systems with mainly hard-bottom habitats along the shores. Coastal parallel water currents predominate in the study area, but situations with transport from or towards the coast also occur. The currents are usually wind-driven, but sometimes caused by water level differences between the ends of the Kalmar Strait, with typical current velocities being 0.1–0.2 m/s [4]. Due to the thermal discharge from the power plant, the gradient coastline habitat between the two bays had a gradual decrease in temperature from the heated bay such that ambient temperatures were reached prior to the entrance to the control bay (**Fig. S8**). It consisted mainly of east-facing wave- exposed rocky shores but also included some relatively sheltered sand bottomed bays.

To quantify spatial variation of wave exposure for the different sites within the gradient, data on coastal wave exposure, modelled using Simplified Wave Model (SWM) [5], was extracted from the Swedish Agency for Marine and Water Management database [6]. In brief, the SWM model estimates wave exposure (at 10 × 10 m resolution) by calculating the distance over open water that wind can travel toward each coastal point from multiple directions, weighting these distances by prevailing wind conditions, and combining them into a long-term index reflecting the relative strength of wave forces. The wave exposure values for the different sites in the gradient environment ranged from a low of 2713 to a high of 232297 m^2s^{-1} (meters squared per second), corresponding to extremely sheltered and moderately exposed habitats, respectively [7]. Unlike water temperature, the variation in wave exposure among sites within the gradient was not correlated with distance from the heated bay (r = 0.03, n = 9, p = 0.93).

Within the three environments, sampling sites were selected to represent the spatial heterogeneity of conditions, subject to the constraints of proximity and accessibility. Eight sampling sites were selected in each bay and eight to ten in the gradient (**Fig. S2)** (see below for details).

The cooling water intake of the OKG power plant was situated in nearby open coastal waters at a depth of 16-18 meters below the sea surface. The sea water was heated to a temperature approximately 10 °C above the ambient water temperature while it cools down the reactors, without being in direct contact with the reactors or any radioactivity [8]. Previous studies

have indicated that the microbial communities in the heated bay were not significantly influenced by microbes arriving to the bay with the inflowing reactor cooling water [8].

Collection of biofilm samples from natural stones

To enable quantification and comparisons of spatiotemporal variation of biofilm communities, samples were collected from naturally occurring stones on six occasions in 2021 (June and October) and 2022 (January, April, July, and October). For this, eight sampling sites in each of the three environments were initially selected (see **Fig. S2** for positions and **Table S1** for coordinates**).** On the first sampling event in June 2021, two samples from different depths were collected from each site (one shallower, 43±12 cm and one slightly deeper, 78±14 cm) (**Table S1**), to investigate whether community composition varied more among different sampling sites than between depths within sampling sites. From October 2021 and onwards, stones were only sampled from the shallower water, and the number of sampling sites in the gradient was increased from 8 to 10 (GI and GJ were added) to better capture the non-linear temperature cline. One sample from October 2021 in the control bay (site CA) was lost and one sampling site in the heated bay (WG) had to be omitted from January 2022 due to the construction of a water pump that prevented access to the site, resulting in a total of 173 samples (**Table S2**).

In the field, biofilm samples were collected from a standardized circular area of 20 cm² from each stone at each of the sampling sites and sampling events. This was achieved by applying a sterile rubber O-ring directly to the surface of the stone and scraping the biofilm from the surface within the O-ring using a sterile toothbrush (ICA Basic medium) and a sterile metal spatula, for ca. 10 mins. The biofilm from each stone was flushed with sterile Milli-Q ultrapure water into a 50 mL sterile Falcon tube (Thermo Scientific™). The samples were maintained on ice during transportation and afterwards frozen at -20 °C at Linnaeus University in Kalmar until further processing.

Collection of biofilms from HOBO-loggers in deeper waters

The biofilm samples from naturally occurring stones described above were collected exclusively from coastal shallow (<1 m) waters. To increase inference space, whether and how long-term warming influences biofilm diversity and community composition was also investigated in deeper waters. To this end, biofilm samples were obtained from HOBO-loggers that had been submerged in the heated and the control bay for one year at a depth of approximately 4±1.4 m (see **Fig. S2** for positions and **Table S3** for individual depths and coordinates). As part of a related sediment translocation study [9, 10], eight metal cages were submerged in each bay at specific sites in August 2021 and recovered one year later in September 2022, except for one cage at site N1, which was lost (see **Fig. S2**). Each cage was equipped with two HOBO-loggers, one situated in proximity to the sediment surface and the other positioned approximately 40 cm above. This resulted in a total of 16 HOBO-logger samples from eight sites in the heated bay and 14 samples from seven sites in the control bay. Biofilm samples were obtained from each HOBO-logger by scraping them individually with a sterile metal spatula and sterile toothbrushes and rinsing with Milli-Q ultrapure water into sterile 50-mL Falcon tubes. The samples were maintained on ice during transportation and

afterwards frozen at -20 °C at Linnaeus University in Kalmar until further processing. Due to insufficient amplification of DNA from the lower HOBO-logger samples (for 7/8 lower samples in the heated bay and for 4/7 lower samples in the control bay), only data from the upper HOBO-loggers were used for the statistical analysis.

Water chemistry

To investigate whether and how water chemistry influenced biofilm composition, water samples were collected monthly from October 2021 to September 2022. The analysis of silicon (Si in mg/L) was conducted by ALS Scandinavia AB in Luleå, Sweden. The lowest concentration limit that was reported (LOR) was set at 0.03 mg/L, and the method employed was the determination of metals and trace elements in water by inductively coupled plasma-atomic emission spectrometry (ICP-AES) in accordance with the standards set forth in SS-EN ISO 11885:2009 and US EPA Method 200.7:1994. Prior to analysis, samples were acidified with 1 mL of high-purity nitric acid per 100 mL of sample. The analysis of phosphorus (in mg/L) was conducted by ALS Czech Republic (Prague, Czech Republic). The LOR was set at 0.03 mg/L, and the method employed was the determination of total phosphorus through discrete spectrophotometry, with the calculation of phosphorus as P_2O_5 and PO_4^{3-} based on the measured values. The nitrogen content (in mg/L) was analyzed by ALS Denmark A/S (Humlebæk, Denmark). The LOR was 0.02 mg/L, and the method employed was oxidative digestion with potassium peroxodisulfate and spectrophotometric measurement.

To evaluate the influence of additional abiotic environmental factors, temperature, oxygen, and conductivity were recorded at each sampling site on each sampling of the natural stones between June 2021 and February 2024 (except June 2022 for oxygen due to probe malfunction). This was done using a multimeter (WTW MultiLine® Multi 3620 IDS, with the electrodes; for pH: SenTix® 940 pH electrode; for conductivity: TetraCon® 925 conductivity cell; for Oxygen: FDO® 925 optical DO sensor). Measurements were also taken at the HOBO-logger sites during the collection of the biofilm in September 2022.

Biofilm sample processing, DNA extraction, amplification and sequencing

The biofilm samples were processed prior to DNA extraction, PCR amplification of the 16S and 18S rRNA genes, and subsequent sequencing. The samples were thawed at room temperature (dark conditions), and the sample volumes subsequently adjusted to 50 mL using Milli-Q ultrapure water. The samples were homogenized by adding seven sterile metal beads (5.5 mm, mpbio) to each sample with a sterile metal spoon and then shaken twice at 5.5 m/sec for 30 seconds in the FastPrep-24™ 5G (MP Biomedicals, LLC) shaker. The metal beads were then removed from each sample with a sterile metal spoon, and a 4 mL subsample was transferred to a 15 mL Falcon tube for DNA extraction.

The 4 mL subsamples taken for DNA extraction were centrifuged for 10 min at 3200 g. The liquid was carefully removed and DNA extraction was performed on the pellet using the ZymoBIOMICS DNA Miniprep Kit (BioSite Nordic) following the manufacturer's instructions. DNA purity was assessed using a Thermo Scientific™ NanoDrop 2000 spectrophotometer (Thermo Fisher Scientific™) and DNA concentration was measured using

an Invitrogen Qubit 2.0 fluorometer (Thermo Fisher Scientific™). For samples with DNA concentrations less than 2 ng/µL, a second DNA extraction was performed using a 10 mL subsample of the homogenized sample. DNA concentrations were normalized to 2 ng/µL by adding molecular grade UltraPure water (BioSite Nordic).

For both 16S and 18S rRNA genes a two-step PCR amplification protocol was used. In the first step, the V3-V4 hypervariable region of the bacterial 16S and the V4 region of the eukaryotic 18S rRNA gene were amplified using 16S and 18S universal primers with a 5′ end overhang adapter sequence corresponding to Illumina Nextera index primers. For 16S rRNA gene, the 341F (5'-CCTACGGGNGGCWGCAG-3') and 805R (5'-GACTACHVGGGTATCTAATCC-3') [11, 12] primer pair was used. For eukaryotes, the TAReuk454FWD1 (5'-CCAGCASCYGCGGTAATTCC-3') [13] and TAReukREV3_modified (V4 18S Next.Rev; 5'-ACTTTCGTTCTTGATYRATGA-3') [14] primer pairs were used. DNA samples were amplified in 25 µL reactions containing 12.5 µL of 2X Phusion High-Fidelity PCR Master Mix (Thermo Fisher Scientific™), 1.25 µM of each primer and 2 µL of the DNA template, with the final volume adjusted to 25 µL using molecular grade water. Two technical PCR replicates were performed. PCR conditions were: initial denaturation at 98°C for 30 s followed by 20 cycles of denaturation at 98°C for 10 s, annealing at 58°C for 30 sec or 65°C for 1 min (for 16S and 18S respectively) and 72°C for 15 s, with a final elongation step of 72°C for 2 min. A no-template control (RNase/DNase-free water) was included in all PCR amplifications to verify the absence of contamination.

The PCR products were purified using AMPure XP magnetic beads (Becker Coulter, USA), checked on a 1% TAE (Tris-Acetate-EDTA) agarose gel, and quantified using a Qubit fluorometer. The product of the first PCR was then used as template in the second amplification step to attach Illumina Nextera multiplexing indexes (N7/N5) and Illumina sequencing adapters (Illumina, San Diego, CA, USA) using the following protocol: 7-10 µL purified PCR product, 1 µM of each index primer and 25 µL Phusion High-Fidelity PCR Master Mix in a total reaction volume of 50 µl. PCR conditions were: 30 s at 98 °C, followed by 12 cycles of 98 °C for 10 s, 62 °C for 30 s and 72 °C for 5 s, and a final elongation at 72 °C for 2 min. The second step PCR products were purified and quantified as above, and the sample libraries were pooled equimolarly in two batches (96 samples in each pool). The quality of the final library pools was checked on a TapeStation 4150 (Agilent) before sequencing on two Illumina MiSeq v3-600 (2 × 300 bp) runs (16S rRNA gene) and two Illumina NextSeq 2000 P1-600 (2 × 300 bp) runs (18S rRNA gene), with 10% PhiX, at Science for Life Laboratory (SciLifeLab), Sweden.

DNA sequence processing

Raw sequences from both the 16S and 18S rRNA gene sequencing were processed using the nf-core-pipeline ampliseq (v.2.9.0) within Nextflow (v.23.10.1) using the default settings (doi: 10.5281/zenodo.1493841) [15]. The ampliseq pipeline uses DADA2 (v.1.30.0 or v.1.22.0) [16] to infer amplicon sequencing variants (ASVs) and assign taxonomy. Briefly, primers were removed, then sequences were trimmed (forward reads at 269 bp and reverse reads at 209 bp for the 16S rRNA gene, and 280 bp and 260 bp, respectively for the 18S

rRNA gene, reads shorter than this were discarded), denoised, merged, and chimeras removed. DADA2 inherently removes singleton reads (individual reads appearing only once within a sample) during the denoising process, as these are likely sequencing errors. No additional filtering was applied to remove singleton ASVs (ASVs with a total abundance of 1 across the entire dataset) after the ampliseq DADA2 pipeline. Taxonomic classification was performed using the SBDI-GTDB database (Sativa curated 16S GTDB database - Release R07-RS207-1; FigShare doi: 10.17044/scilifelab.14869077.v4) for 16S rRNA gene sequences and the Protist Reference Ribosomal Database (PR^2) v.5.0.0 [17] for 18S rRNA genes. 16S rRNA gene sequences annotated as chloroplast or mitochondria were excluded and for 18S rRNA genes only those annotated as Eukaryota were retained. As micro-eukaryotes are the focus of this study, ASVs assigned to Metazoa (Subdivision = Metazoa) and Embryophyta (Class = Embryophyceae) were removed prior to all downstream analyses. Eukaryotic ASVs were further classified into one of three trophic groups based on their way of obtaining energy: phototrophs, heterotrophs, or mixotrophs. The Mixoplankton Database (MDB, DOI: 10.5281/zenodo.7560582) developed by Mitra et al. [18] was used to identify mixotrophic species. For ASVs belonging to species not found in the MDB, trophic groups were assigned according to taxonomic classification at supergroup, division, subdivision, and class level (see **Table S4**). The October 2021 sample from site CB contained only 3,077 18S rRNA gene reads after sequence processing and was removed from subsequent analyses. The remaining 18S rRNA gene samples had between 28,548 and 1,400,786 counts per sample (average 422,331 ± 232,555) assigned to a total of 41,729 ASVs. The 16S rRNA gene samples had between 42,399 and 241,781 per sample (average 83,574 ± 25,629), assigned to 50,051 ASVs. A complete list of sample names, sequence facility IDs, number of reads obtained before and after running the pipeline, and the associated environmental factors and water chemistry is available in Supplementary **Table S5** while rarefaction curves are provided in **Fig. S3**.

Statistical analyses

Further data analysis was conducted using R (v.4.3.1) and RStudio (v.2023.06.2) [19]. Packages used include vegan (v.2.6-10) for rarefaction curves, permutational Multivariate Analysis of Variance (perMANOVA) and Redundancy Analyses (RDA), lme4 (v.1.1-36) and lmerTest (v.3.1-3) for linear mixed-effects models (LMM), emmeans (v.1.11.0) for pairwise comparisons, and MicEco (v.0.9.19), microViz (v.0.12.6), MicrobiomeStat (v.1.4.1) and ggplot2 for visualizations. In some cases, natural language processing tools driven by AI (Copilot) were used to create or edit the R code.

For alpha diversity analyses, data were rarefied to the lowest sequencing depth within each dataset (16S: 42,399 for June 21 and full stone sample dataset, 76,867 for the HOBO loggers; 18S: 69,369 for June 21, 37,618 for the full stone dataset, and 28,548 for the HOBO loggers) using MicrobiomeStat. Alpha diversity was estimated using the Shannon diversity index in vegan.

To assess the effects of environment and water depth (for the HOBO logger and June 21 samples) or environment (control bay, heated bay, and the gradient) and time (for the full

stone sample dataset) on alpha diversity, linear mixed-effects models (LMM) were fitted using the lmer function from lmerTest. For June 21 samples, the model included environment (control bay, heated bay, gradient), depth (categorical: shallower vs deeper), and their interaction, with site as a random intercept. For the full temporal stone dataset (shallow samples only) the model tested environment, sampling date, and their interaction, with site as a random intercept. While for the HOBO-loggers, a linear model was used that included bay (control vs heated), water depth (continuous variable in meters), and their interaction. Significance was evaluated using Type III Analysis of Variance with Satterthwaite's method (anova function). Pairwise comparisons between sampling occasions within each environment were conducted using emmeans with Tukey adjustment.

To examine the direct relationship between alpha diversity and water temperature across all environments and sampling occasions, additional LMMs were fitted with temperature as a fixed effect and site as a random intercept. For the gradient environment specifically, LMMs were fitted to test the effects of temperature, sampling date, and wave exposure ($\log(m^2s^{-1})$) on alpha diversity, with site included as a random intercept.

Unique and shared ASVs among environments were quantified using the ps_venn function of the MicEco package and visualized using Euler diagrams. ASVs unique to each environment (present in only one environment) were identified and LMMs were run to assess the effects of environment and season (summer: June 2021, July 2022; autumn: October 2021, October 2022; winter: January 2022; and spring: April 2022) on the number of unique prokaryote and micro-eukaryote ASVs, including the interaction between environment and season, with site as a random intercept. Pairwise comparisons were conducted using emmeans with Tukey adjustment to identify differences between environments within each season and between seasons within each environment.

To assess the effects of environment and sampling data on the relative abundance of the eukaryote trophic groups, separate LMMs were fitted for phototrophs, heterotrophs and mixotrophs. The models included environment, sampling date, their interaction and site as a random intercept. To distinguish significant differences across sampling dates in each environment, pairwise post-hoc comparisons with Tukey adjustment were used.

For each environment, the relationships between the relative abundance of each trophic group and all environmental variables were tested using Spearman rank correlations and visualized in a heatmap. To correct for multiple comparisons, a false discovery rate (FDR) adjustment (Benjamini-Hochberg method) was applied.

For analyses of beta diversity, Euclidean distance matrices of robust centred-log ratio (rCLR)-transformed data (i.e., Aitchison distances) were constructed using the decostand function in vegan. Aitchison distances were used to appropriately account for the compositional nature of microbiome data. Differences in the prokaryote and micro-eukaryote community composition (beta diversity based on Aitchison distance) across the different environments, sampling depths, and time were visualized through Principal coordinate

analysis (PCoA) using cmdscale. Differences in community composition between communities in the three environments, with water depth, and across sampling dates were tested using permutational multivariate analysis of variance (perMANOVA) using the adonis2 function in vegan with 9999 permutations. For the June 2021 dataset, perMANOVA models tested the effects of environment (categorial: control bay, heated bay, and gradient) and water depth (categorial: shallower vs slightly deeper) and their interaction. For the full temporal dataset (shallow stones), the models tested environment, sampling date (categorial), and their interaction. For the gradient environment, the effects of temperature, sampling date, and wave exposure were tested, and for the HOBO-loggers, the models tested bay (control bay vs heated bay), water depth (in meters, continuous), and their interaction. Homogeneity of multivariate dispersions was assessed using the betadisper vegan function.

To assess whether single-stone sampling per site was sufficient to capture site-level patterns despite potential micro-scale heterogeneity in biofilm communities, the June 2021 dataset was analyzed when two stones (shallower and slightly deeper) were collected at each site. perMANOVA (adonis2 function in vegan, 9999 permutations) was used to partition variance in community composition (based on Aitchison distances) into components explained by site identity versus residual within-site variation (including depth and micro-scale patchiness). This analysis was performed separately for prokaryotes and micro-eukaryotes, both across all three environments combined (model: ~ Bay + Site) and within each environment individually (model: ~ Site), to quantify the proportion of total variance (R^2) attributable to site-level differences.

To investigate the relationship between the investigated environmental factors and the community composition of the biofilm, RDAs were performed on rCLR-transformed data to account for the compositional nature of the data. Predictor variables were cantered and scaled prior to analysis. For the June 2021 dataset, environmental variables included water depth, temperature, conductivity, and oxygen. For the full temporal dataset (shallow stones), variables included temperature, conductivity, oxygen, silicon, phosphorus, and nitrogen. For the gradient environment, wave exposure (m^2s^{-1}, modelled using SWM) was included as an additional predictor. For HOBO-loggers, the variables included biofilm depth, temperature, conductivity, and oxygen.

Variance Inflation Factors (VIF) were calculated using vif.cca to assess multicollinearity among the predictor variables; all VIF values were below 5, indicating acceptable collinearity. The significance of each environmental variable was assessed using permutation tests (9999 permutations) with by = "margin" to evaluate the marginal effect of each term. The proportion of total variance explained by each significant predictor was calculated as the variance explained by that term divided by the total inertia.

The perMANOVA and the RDA analyses were used separately to avoid collinearity, as most measured physicochemical variables covary strongly with environment and/or season. The permMANOVA tested categorical experimental design factors (environment, season, depth), while the RDA tested continuous environmental drivers (temperature, conductivity, nutrients,

etc.). This approach allowed quantification of the contribution of both integrated environmental effects (captured by categorical variables) and specific physicochemical drivers to community composition.

Supplementary Results

Dominating taxonomic taxa in biofilm communities sampled in June 2021

In June 2021, for prokaryotes (**Figure S6A**), the most dominating taxonomic taxa (classes) were Cyanobacteria in the heated bay and the gradient, followed by Alphaproteobacteria. In the control bay, the dominating groups were Cyanobacteria, Alphaproteobacteria, Gammaproteobacteria, and Bacteroidia. For micro-eukaryotes (**Fig. S6B**), the dominating taxonomic taxa were Phaeophyceae, Bacillariophyceae, Ulvophyceae and Florideophyceae, with a large number of unknown classes. For both prokaryotes and micro-eukaryotes, the relative abundance of dominant classes varied considerably among sites within environments.

Dominating taxonomic taxa in biofilm communities sampled in different sampling sites, environments, and sampling occasions from October 2021 to October 2022

The data indicated that the domination of taxonomic classes were not uniformly distributed across the sampling sites, environments, and sampling occasions. For prokaryotes, the Alphaprotepbacteria, Cyanobacteria, and Gammaproteobacteria were generally most abundant, with Bacteroidia showing high relative abundance in April 2022, particularly in the gradient (**Fig. S9**). For the micro-eukaryotes, Ulvophyceae and Florideophyceae were abundant in all three environments, whereas other classes were more variable in relative abundance across seasons and between environments. Phaeophyceae were more abundant in the gradient and the control bay than in the heated bay, Bacillariophyceae were more abundant in the gradient than in the two bays, and Chrytridiomycota were more abundant in the heated bay (**Fig. S10**).

Dominating taxonomic taxa in biofilm communities sampled from HOBO loggers incubated for one year at deeper waters in the heated and control bay

The results from the analysis of the biofilm samples obtained from HOBO-loggers that had been submerged in the heated and the control bay for one year at a depth of approximately 4±1.4 m as part of a related sediment translocation study, informed about dominating taxa at deeper waters. The prokaryotic biofilm community sampled at deeper waters was in general dominated by Cyanobacteria, Alphaproteobacteria, Gammaproteobacteria, and Planctomycetia both in the control and the heated bay (**Fig. S12A**). For the eukaryotes, the classes Bacillariophyceae, Phaeophyceae, Ulvophyceae, Chlorophyceae, and Dinophyceae dominated in the control bay, but with considerable variation among sites. In the heated bay, the dominating micro-eukaryotic classes were Florideophyceae, Bacillariophyceae, Oligohymenophorea, Coscinodiscophyceae, Trebouxiophyceae, and Gregarinomorphea, again with considerable site dependence (**Fig. S12B**). While sequences assigned to Metazoa were excluded from the analyses prior to filtering, these included Bryozoa that accounted for 23 % of the HOBO-logger sample reads (compared to 0.5 % in the stone samples), which was consistent with the visual observation that Bryozoa dominated the HOBO-logger surface.

Supplementary Tables

Table S1. Depth of natural stones found in slightly deeper water (June 2021), average depth of natural stones in shallower waters (entire study period) for each site, and the coordinates.

Environment	Site	Deep depth (cm)	Shallow depth (cm)	Latitude	Longitude
Control bay	CA	89	51	N 57°25.988	E 16°40.913
	CB	93	48	N 57°25.989	E 16°40.306
	CC	90	46	N 57°25.944	E 16°40.734
	CD	91	52	N 57°26.032	E 16°41.284
	CE	90	51	N 57°26.056	E 16°41.602
	CF	86	53	N 57°26.132	E 16°41.799
	CG	77	40	N 57°26.133	E 16°41.799
	CH	82	52	N 57°25.945	E 16°40.845
Heated bay	WA	94	48	N 57°25.112	E 16°40.684
	WB	83	46	N 57°25.116	E 16°40.765
	WC	96	49	N 57°25.098	E 16°40.861
	WD	87	42	N 57°25.094	E 16°40.955
	WF	84	37	N 57°25.126	E 16°40.609
	WG	81	63	N 57°25.105	E 16°40.307
	WH	79	45	N 57°25.385	E 16°40.062
	WI	91	49	N 57°25.330	E 16°39.929
Gradient	GA	55	43	N 57°25.314	E 16°41.797
	GB	71	42	N 57°25.619	E 16°41.816
	GC	70	41	N 57°25.515	E 16°41.641
	GD	51	32	N 57°25.415	E 16°41.572
	GE	60	37	N 57°25.652	E 16°41.394
	GF	68	40	N 57°25.300	E 16°41.221
	GG	51	26	N 57°25.211	E 16°41.020
	GH	55	38	N 57°25.148	E 16°40.990
	GI	NA	32	N 57°25.259	E 16°41.029
	GJ	NA	37	N 57°25.189	E 16°42.033

Table S2. Number of biofilm samples collected from natural stones in the gradient and the control and heated bays on each sampling date.

		2021		2022			
Water depth	Environment	June	October	January	April	July	October
Shallow (43 ± 12 cm)	Control	8	7 (8)	8	8	8	8
	Gradient	8	10	10	10	10	10
	Heated	8	8	7	7	7	7
Deep (78 ± 14 cm)	Control	8	-	-	-	-	-
	Gradient	8	-	-	-	-	-
	Heated	8	-	-	-	-	-

Table S3. Depth for the cages where the HOBO-loggers were on at the time of harvest and the coordinates.

Site	Date	Bay	Depth (m)	Latitude	Longitude
K	2022-09-18	Control	2.7	N 57° 26.016'	E 16°41.019'
N3	2022-09-18	Control	4.6	N 57° 26.117'	E 16°42.000'
J	2022-09-18	Control	6.9	N 57° 25.974'	E 16°41.233'
L	2022-09-19	Control	1.9	N 57° 25.967'	E 16°40.926'
N2	2022-09-19	Control	4.4	N 57° 26.049'	E 16°41.696'
M	2022-09-19	Control	3.3	N 57° 25.896'	E 16°41.029'
I	2022-09-19	Control	5.3	N 57° 25.950'	E 16°41.681'
H2	2022-09-23	Heated	4.9	N 57° 25.140'	E 16°40.440'
B	2022-09-23	Heated	3.8	N 57° 25.268'	E 16°40.131'
C2	2022-09-23	Heated	3.6	N 57° 25.318'	E 16°40.042'
F	2022-09-23	Heated	2.1	N 57° 25.210'	E 16°39.907'
E	2022-09-23	Heated	2.1	N 57° 25.198'	E 16°39.774'
A	2022-09-24	Heated	5.5	N 57° 25.134'	E 16°40.265'
A2	2022-09-24	Heated	4.3	N 57° 25.134'	E 16°40.237'
C	2022-09-24	Heated	3.4	N 57° 25.300'	E 16°39.996'

Table S4. Micro-eukaryote trophic group classification. ASVs were classified into one of three trophic groups based on their way of obtaining energy: phototrophs, heterotrophs, or mixotrophs. The Mixoplankton Database (MDB, DOI: 10.5281/zenodo.7560582) [18] was used to identify mixotrophic species (251 ASVs were assigned as mixotrophs - mainly dinoflagellates). For ASVs belonging to species not found in MDB, trophic groups were assigned according to the below taxonomic classification.

Trophic Group	Taxonomic Level	Taxa assigned
Phototroph	Division	Chlorophyta, Rhodophyta, Streptophyta, Prasinodermophyta, Cryptophyta:nucl
	Subdivision	Chrompodellids
	Class	Bacillariophyceae, Bolidophyceae, Coscinodiscophyceae, Eustigmatophyceae, Mediophyceae, Phaeothamniophyceae, Phaeophyceae, Raphidophyceae, Xanthophyceae, MOCH-2, MOCH-3, MOCH-5, Pelagophyceae, Chrysomerophyceae, Dictyochophyceae, Synchromophyceae
Heterotroph	Supergroup	Amoebozoa, CRuMs, Obazoa, Provora
	Division	Kathablepharidacea, Ancyromonadida, Hemimastigophora, Centroplasthelida, Rhizaria, Picozoa, Telonemia, Metamonada
	Subdivision	Discoba_X, Apicomplexa, Ciliophora, Colponemidia, Perkinsea, Bigyra
	Class	Gyrista_X, Hyphochytriomyceta, Peronosporomycetes, Pirsoniales, Developea
	Genus	Rhodelphis
Mixotroph	Division	Haptophyta, Cryptophyta
	Subdivision	Dinoflagellata, Euglenozoa
	Class	Chrysophyceae
	Genus	Phaeodactylum

Table S5. Sequencing sample data. List of sample names, environmental and water chemistry metadata, sequence facility IDs, number of reads obtained before and after merging paired-end reads, denoising and taxonomic filtering.

Provided as an additional xlsx file.

Table S6. Statistics for the linear mixed-effects models assessing the effects of environment and water depth (for the HOBO logger and June 21 samples) or environment and sampling date (Season, for the full shallow stone sample dataset) on alpha diversity for both prokaryotes and micro-eukaryotes. The models included interaction terms and site as a random intercept. Significance was evaluated using Type III Analysis of Variance with Satterthwaite's method.

Alpha diversity		Prokaryotes			Micro-eukaryotes		
Experiment	Parameter	NUMDF; DENDF	*F*	*p*	NUMDF; DENDF	*F*	*p*
June 2021	Environment	2; 48.8	4.63	**0.014**	2; 74.65	5.46	**0.006**
June 2021	Depth	1; 105.68	4.14	**0.043**	1; 107.09	9.49	**0.003**
June 2021	Environment x Depth	2; 105.58	3.23	**0.043**	2;106.90	2.12	0.126
Shallow stones	Environment	2; 131	2.08	0.129	2; 22.25	3.86	**0.036**
Shallow stones	Season	5; 131	2.55	**0.031**	5; 108.36	2.58	**0.030**
Shallow stones	Environment x Season	10; 131	2.85	**0.003**	10; 108.30	2.77	**0.004**
HOBO-loggers	Environment	1; 11	0.82	0.384	1; 11	0.13	0.729
HOBO-loggers	Depth	1; 11	0.39	0.545	1; 11	0.33	0.578
HOBO-loggers	Environment x Depth	1; 11	0.06	0.804	1; 11	0.19	0.675

Table S7. Statistics for the perMANOVA based on Aitchison distances showing associations of biofilm community composition with environment (heated bay, control bay, and gradient), water depth, and sampling data (season, for shallow stones only). Results from separate analyses of prokaryotes and micro-eukaryotes, using data from three different sample collections ('experiments').

perMANOVA		Prokaryotes				Micro-eukaryotes			
Experiment	Parameter	NUMDF ;DENDF	R^2	*F*	*p*	NUMDF ;DENDF	R^2	*F*	*p*
June 2021	Environment	2; 42	0.66	45.47	**0.001**	2; 42	0.69	47.91	**0.001**
June 2021	Depth (shallower vs deeper)	1; 42	0.02	2.53	0.076	1; 42	0	0.57	0.587
June 2021	Envir. x Depth	2; 42	0.02	1.61	0.170	2; 42	0.01	0.51	0.744
Shallow stones	Environment	2; 131	0.19	25.2	**0.001**	2; 130	0.52	96.71	**0.001**
Shallow stones	Season	5; 131	0.17	9.09	**0.001**	5; 130	0.09	6.97	**0.001**
Shallow stones	Envir. x Season	10; 131	0.13	3.42	**0.001**	10; 130	0.04	1.51	0.061
HOBO-loggers	Bay	1; 15	0.47	21.6	**0.001**	1; 11	0.51	15.66	**0.001**
HOBO-loggers	Depth (cm)	1; 15	0.26	12	**0.015**	1; 11	0.1	2.97	0.061
HOBO-loggers	Bay x Depth	1; 15	0.02	1.07	0.371	1; 11	0.03	0.94	0.419

Table S8. Statistics for the alpha and beta diversity analyses in the gradient. Linear mixed-effects model assessing the effects of temperature, wave exposure, and sampling occasion on alpha diversity; perMANOVA assessing the effects of temperature, wave exposure and sampling date on Aitchison distances; and RDA analyses on rCLR transformed data assessing the relative influence of temperature wave exposure, conductivity, oxygen, silicon, phosphorus and nitrogen.

**Alpha diversity**	Prokaryotes					Micro-eukaryotes				
LMM parameter	NUMDF; DENDF	SumSq	MeanSq	*F*	*p*	NUMDF; DENDF	SumSq	MeanSq	*F*	*p*
Temperature	1; 58	3.99	3.99	7.39	**0.009**	1; 24.30	0.04	0.04	0.06	0.806
Samping date	5; 58	11.63	2.33	4.31	**0.002**	5; 43.48	14.69	2.94	4.60	**0.002**
Wave exposure	1; 58	0.65	0.65	1.21	0.277	1; 6.65	0.00	0.00	0.00	0.955

**Beta diversity**										
perMANOVA parameter	NUMDF; DENDF	SumSq	R^2	*F*	*p*	NUMDF; DENDF	SumSq	R^2	*F*	*p*
Temperature	1; 57	1194.38	0.02	2.47	0.080	1; 57	1708.05	0.05	6.07	**0.003**
Sampling date	5; 57	18479.22	0.31	7.65	**0.0001**	5; 57	9828.78	0.28	6.99	**0.0001**
Wave exposure	1; 57	6604.56	0.11	13.67	**0.0001**	1; 57	8439.55	0.24	29.99	**0.0001**

RDA parameter	NUMDF; DENDF	Variance	Variance (%)	*F*	*p*	NUMDF; DENDF	Variance	Variance (%)	*F*	*p*
Temperature	1; 32	6.46	0.64	0.46	0.696	1; 32	9.82	1.45	1.06	0.343
Wave exposure	1; 32	100.17	9.95	7.21	**0.001**	1; 32	86.78	12.83	9.37	**0.002**
Conductivity	1; 32	22.56	2.24	1.62	0.208	1; 32	8.96	1.32	0.97	0.374
Oxygen	1; 32	94.72	9.41	6.81	**0.002**	1; 32	71.28	10.54	7.70	**0.002**
Silicon	1; 32	34.76	3.45	2.5	0.081	1; 32	24.61	3.64	2.66	0.066
Phosphorus	1; 32	89.50	8.89	6.44	**0.001**	1; 32	30.30	4.48	3.27	**0.040**
Nitrogen	1; 32	22.43	2.23	1.61	0.183	1; 32	22.13	3.27	2.39	0.100

Supplementary Figures

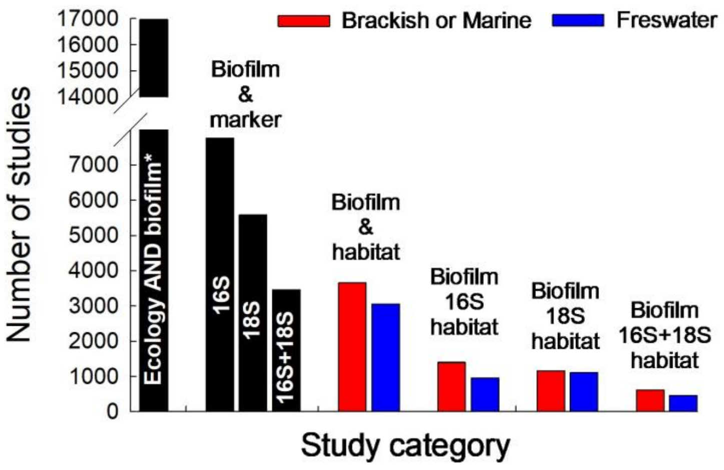

Figure S1. Trends in absolute research output on studies of the ecology of biofilms that used different rRNA molecular markers (16S, 18S or both) to study prokaryotic and eukaryotic taxa in brackish and marine or freshwater habitats. The figure shows the total number of publications up to June 30, 2025. Note that the vertical axis contains a break to improve resolution of lower values.

The figure is based on data extracted from topic searches conducted on 15 September 2025 in ISI Web of Science (Data base: all; time span: 1900-01-31 - 2025-06-30) using the following search strings:

0. Biofilm*, generated 168790 papers (not shown).
1. Ecology AND biofilm*, generated 16964 papers. 2. Ecology AND biofilm* AND (16S OR prokaryot*), generated 7760 papers.
3. Ecology AND biofilm* AND (18S OR eukaryot*), generated 5581 papers.
4. Ecology AND biofilm* AND (16S OR prokaryot*) AND (18S OR eukaryot*), generated 3460 papers.
5. Ecology AND biofilm* AND (brackish OR marine), generated 3652 papers.
6. Ecology AND biofilm* AND freshwater*, generated 3056 papers.
7. Ecology AND biofilm* AND (16S OR prokaryot*) AND (brackish OR marine), generated 1399 papers.
8. Ecology AND biofilm* AND (16S OR prokaryot*) AND freshwater, generated 963 papers.
9. Ecology AND biofilm* AND (18S OR eukaryot*) AND (brackish OR marine), generated 1158 papers.
10. Ecology AND biofilm* AND (18S OR eukaryot*) AND freshwater, generated 1109 papers.
11. Ecology AND biofilm* AND (16S OR prokaryot*) AND (18S OR eukaryot*) AND (brackish OR marine), generated 613 papers.
12. Ecology AND biofilm* AND (16S OR prokaryot*) AND (18S OR eukaryot*) AND freshwater, generated 459 papers.
13. Ecology AND biofilm* AND (16S OR prokaryot*) AND (18S OR eukaryot*) AND (brackish OR marine) AND “Baltic Sea”, generated 6 papers (not shown).

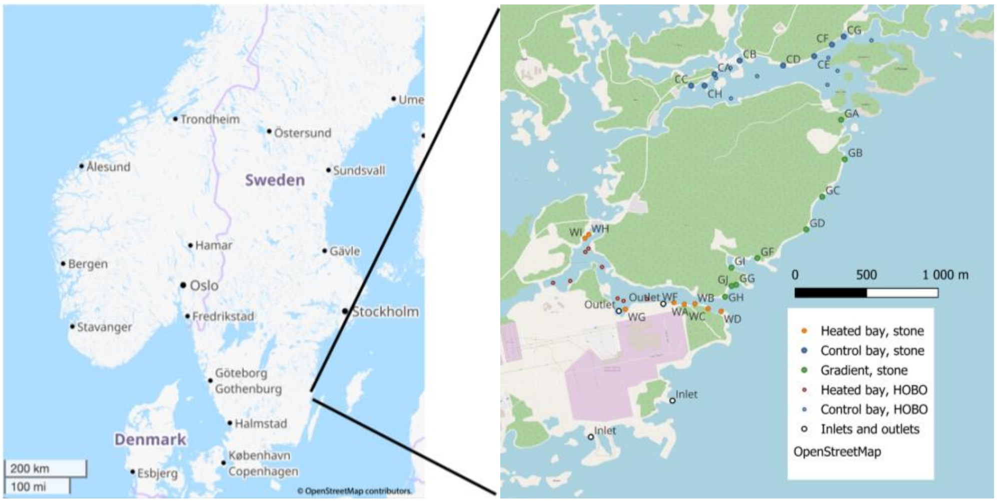

Figure S2. Map of study area showing the locations where biofilm samples were collected from natural stones and HOBO-loggers. Orange represents the heated bay, green the gradient, and blue the control bay. The darker color shade represents where the stones were taken, the lighter color shades where the deeper cages with the HOBO-loggers were placed, and the white dots represent the water inlet and outlet. The sites for the stones are marked with the name of the site (see coordinates in Table S1) while the HOBO-logger coordinates can be found in **Table S3**. The map was generated in QGIS using the OpenStreetMap base map, licensed under CC BY_SA 2.0. For information on spatial and temporal temperature variation in the different environments see **Figure S7**.

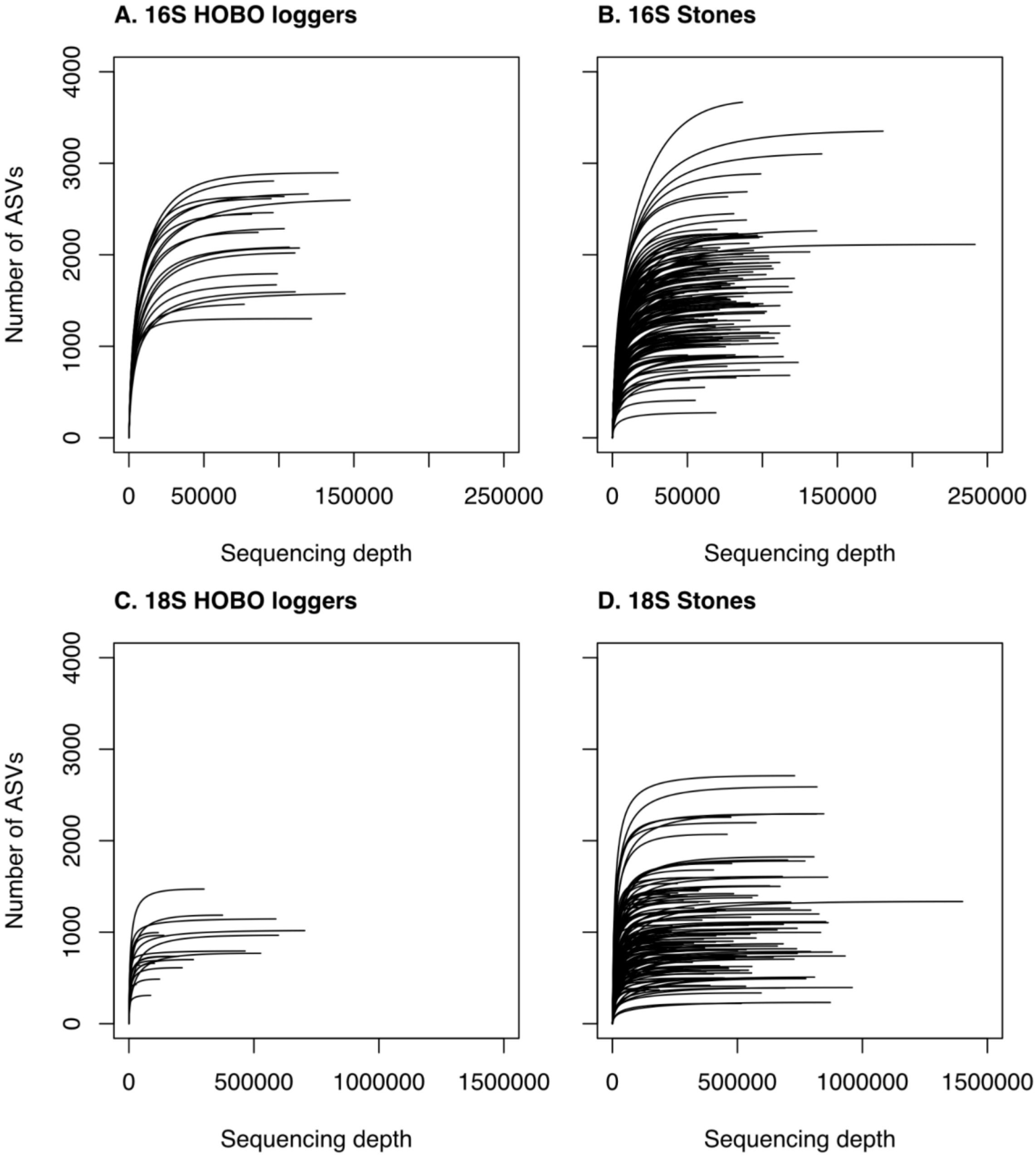

Figure S3. Rarefaction curves. Data for the (A) prokaryote HOBO logger samples (*n* = 19), (B) prokaryote stone samples (*n* = 149), (C) micro-eukaryote HOBO logger samples (*n* = 19) and (D) micro-eukaryote stone samples (*n* = 148).

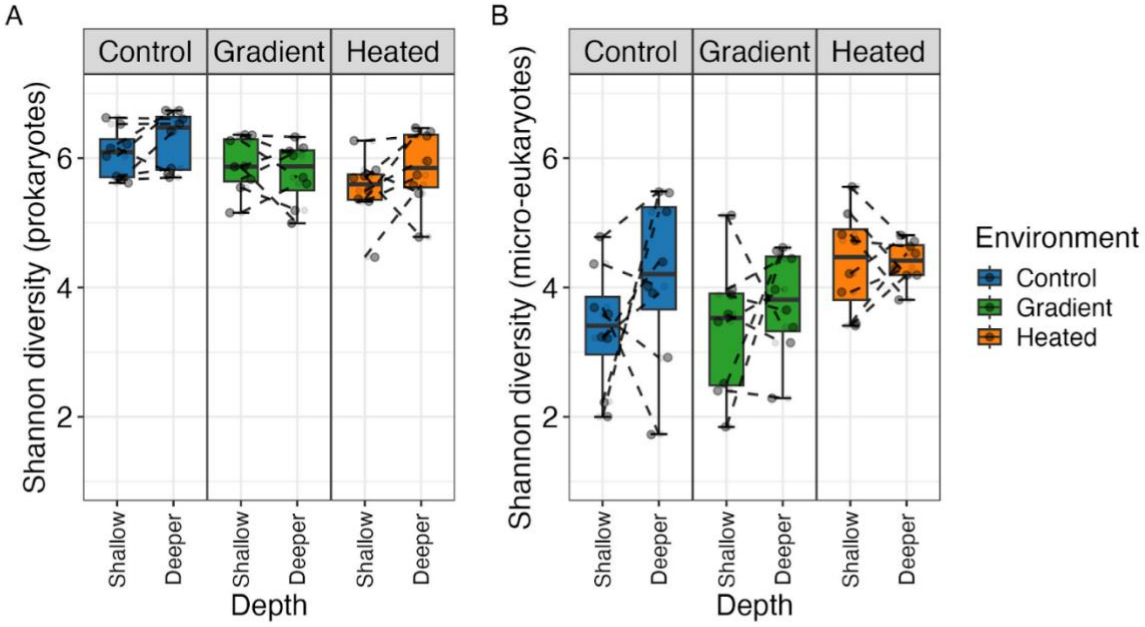

Figure S4. Relationship between Shannon diversity index and water depth in the three different environments in June 2021 for prokaryotes (A) and micro-eukaryotes (B). Blue represents the control bay, green represents the gradient, and orange represents the heated bay. Dashed lines connect shallower (43±12 cm) and slightly deeper (78±14 cm) samples taken from the same site. For prokaryotes there was a significant interaction between water depth and environment ($F_{2,105}$=3.22, p=0.043; **Table S6**) while for micro-eukaryotes there were only significant main effects of the environment ($F_{2,75}$=5.46, p=0.006) and water depth ($F_{2,107}$=9.49, p=0.002; **Table S6**)

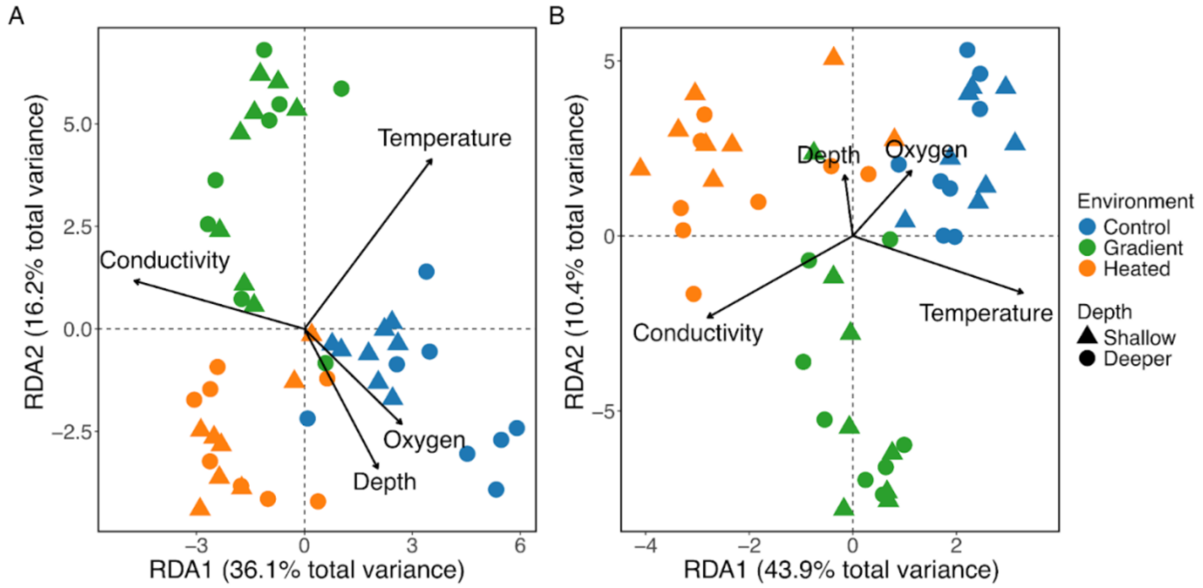

Figure S5. Redundancy analysis (RDA) ordination plots on the rCLR-transformed data from June 2021 for prokaryotes (A) and micro-eukaryotes (B). The arrows indicate the direction and strength of the relationships. The points represent individual samples, orange represents the heated bay, blue the control bay, and green the gradient. Samples from slightly deeper waters (78±14 cm) are shown with a circle and from shallower water (43±12 cm) are shown with a triangle. Temperature, conductivity, and water depth were significant predictors for prokaryotic community composition, while for micro-eukaryotes only temperature and conductivity were significant (**Table 1**).

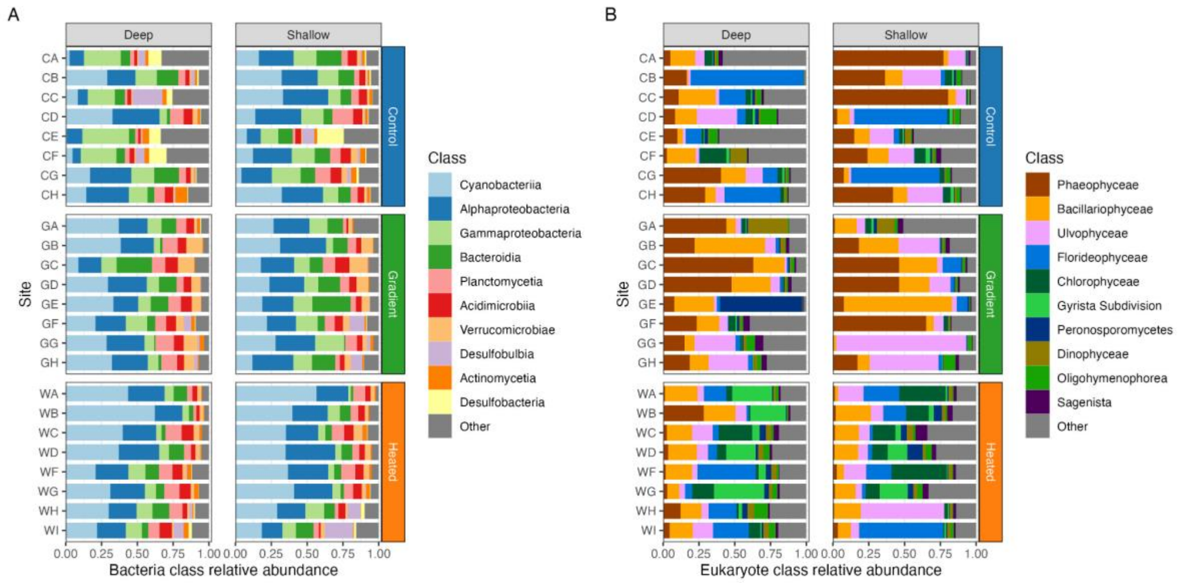

Figure S6. Relative abundance of the ten most dominant classes in the biofilm communities on naturally occurring stones in June 2021 for prokaryotes (A) and micro-eukaryotes (B) in different environments (heated bay, gradient, and control bay). Each bar represents one sample, with samples taken from both shallower (43±12 cm) and deeper waters (78±14 cm) at each site.

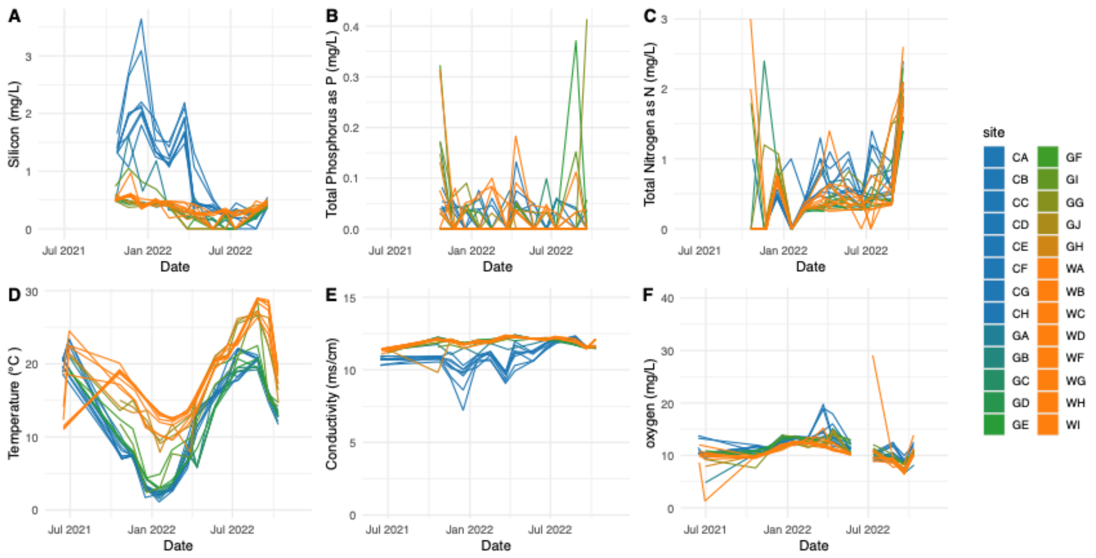

Figure S7. Spatial and temporal variation in water chemistry in the heated bay (orange), the control bay (blue) and the gradient (green) during the period June 2021 to October 2022. Panel A shows silicon, B for phosphorus, C for nitrogen, D for temperature, E for conductivity, and F for oxygen. Missing data for oxygen was due to a broken probe. The locations of the different sampling sites are shown in **Figure S2**.

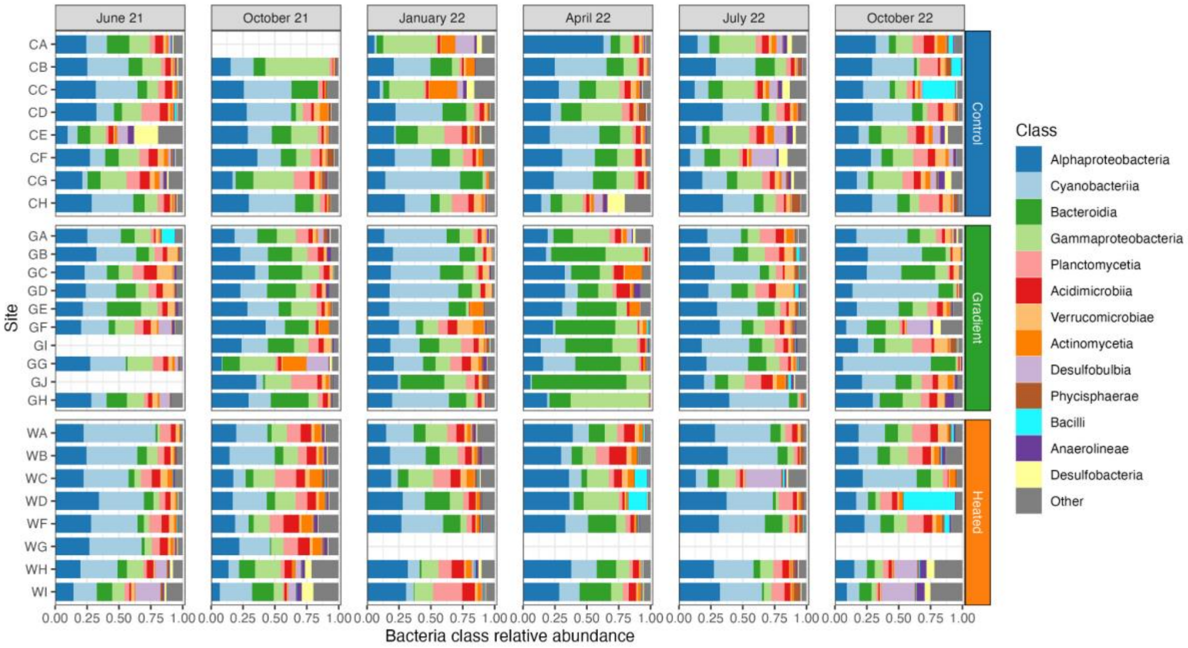

Figure S8. Variation in relative abundance of the prokaryotic classes found in the biofilm on naturally occurring stones from June 2021 until October 2022 at the different sites in the two bays and the gradient between the two bays. Each bar represents one sample while sites within each of the environments are shown on the y-axis and the relative abundance on the x-axis.

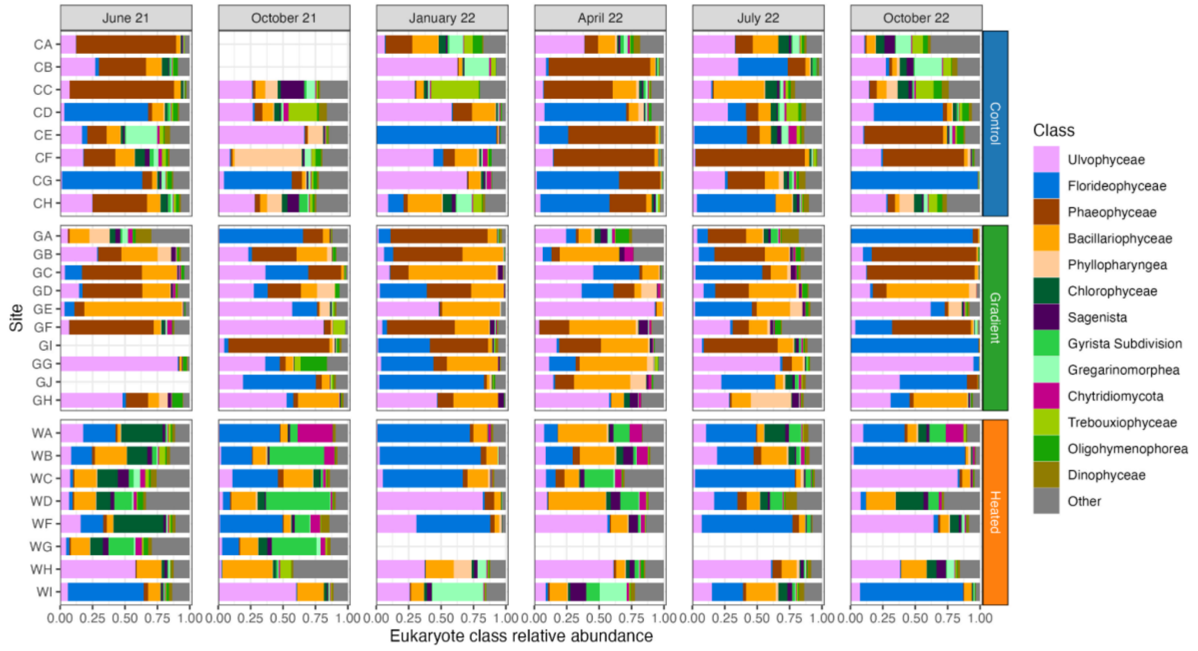

Figure S9. Variation in relative abundance of the micro-eukaryotic classes found in the biofilm on naturally occurring stones from June 2021 until October 2022 at the different sites in the two bays and the gradient between the two bays. Each bar represents one sample while sites within each of the environments are shown on the y-axis and the relative abundance on the x-axis.

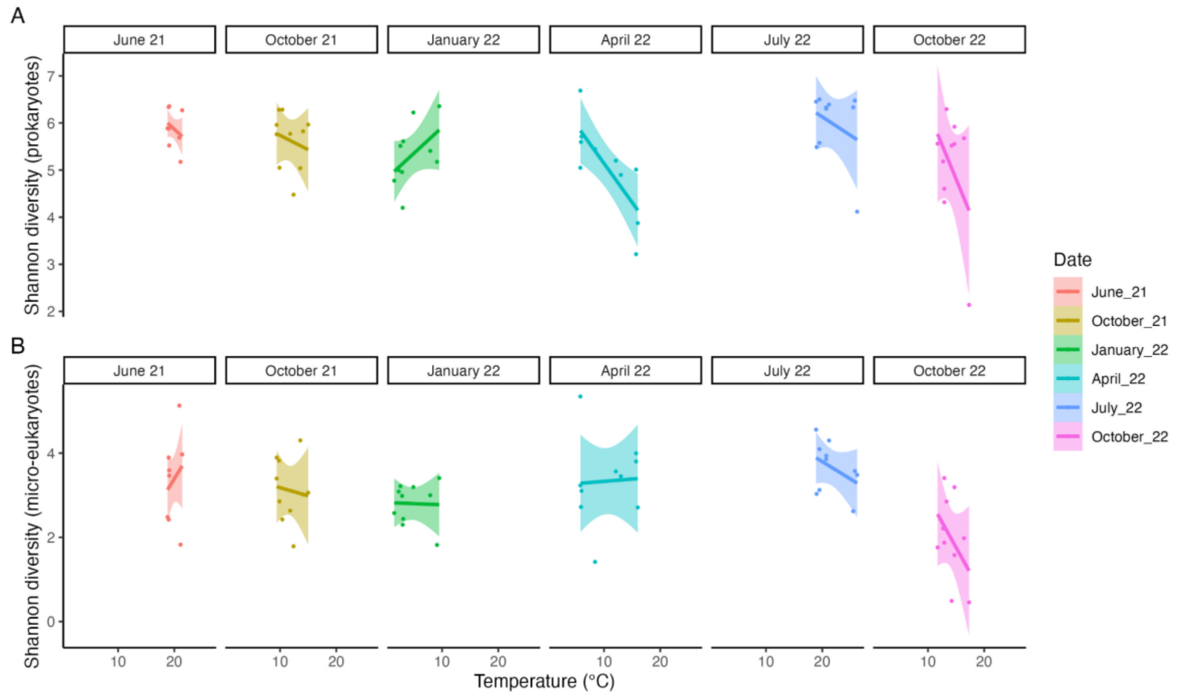

Figure S10. Relationship between water temperature and the alpha diversity of prokaryotes (A) and micro-eukaryotes (B) for different sampling occasions in the gradient. Each color symbolizes a specific date.

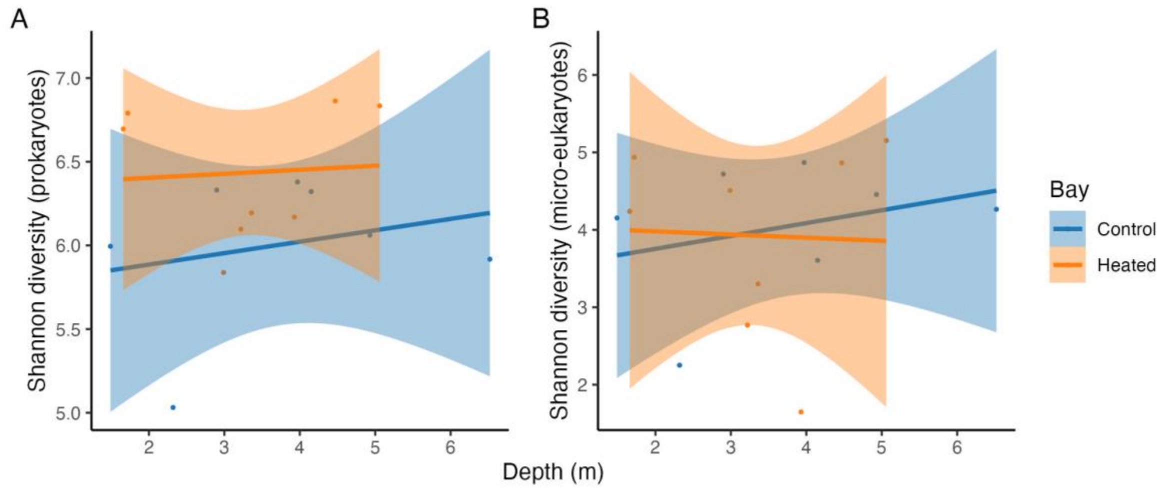

Figure S11. Shannon diversity of prokaryotes (A) and micro-eukaryotes (B) in relation to water depth in samples of biofilm collected from HOBO-loggers maintained for one year in the control bay (blue) or the heated bay (orange). Shannon index is on the y-axis and water depth is on the x-axis. The relationship of diversity with water depth was not statistically significant and also did not differ significantly between bays (**Table S6**).

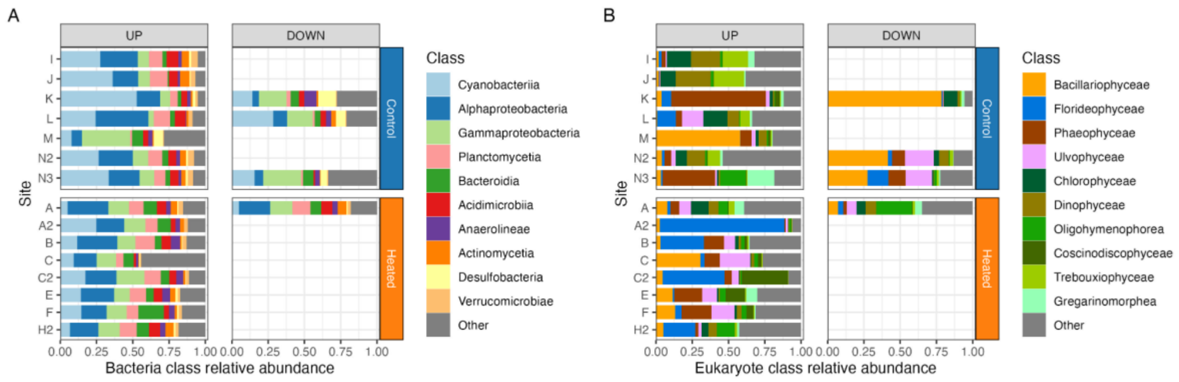

Figure S12. Relative abundance of prokaryotes (A) and micro-eukaryotes (B) found in the biofilm taken from the HOBO-loggers maintained for one year at different sites in the heated and the control bay. Each bar represents one sample. The sites in the two bays are shown on the y-axis and the relative abundance on the x-axis. Up and down indicates the results for the HOBO-loggers that were placed in the upper and the lower positions of the experimental units.

Supplementary References

1. Luo C, Guo L, Zeng S, Long T. Effects of turbulence fluctuation intensity in bioreactor of sewage treatment on physical and chemical properties of biofilms. *Bioproc Biosys Engin*. 2021;**44**:1865-74 https://doi.org/10.1007/s00449-021-02566-y
2. Molobela IP, Ilunga FM. Impact of bacterial biofilms: the importance of quantitative biofilm studies. *Ann Microbiol*. 2012;**62**:461-67 https://doi.org/10.1007/s13213-011-0344-0
3. Salgar-Chaparro SJ, Lepkova K, Pojtanabuntoeng T, Darwin A, Machuca LL. Nutrient level determines biofilm characteristics and subsequent impact on microbial corrosion and biocide effectiveness. *Appl Environ Microbiol*. 2020;**86**:e02885-19 https://doi.org/10.1128/AEM.02885-19
4. Ehlin U, Borenäs K, Neuman E, Sandström O. Miljöeffekter av stora kylvattenutsläpp i ett varmare klimat (in Swedish with en extended English summary). Report. 2012
5. Isæus M. Factors structuring *Fucus* communities at open and complex coastlines in the Baltic Sea. Botaniska institutionen, Doctoral Dissertation. Stockholm University, 2004
6. HAV. Fysisk påverkan i svenska kustvatten 1960 och framåt: Vågexponering. (January 29, date last accessed).
7. Albertsson J, Bergström U, Isæus M, Kilnäs M, Mattisson A, Sandman A. *Sammanställning och analys av kustnära undervattensmiljö (SAKU)*: Naturvårdsverket Stockholm, Sweden, 2006.
8. Seidel L, Ketzer M, Broman E, Shahabi-Ghahfarokhi S, Rahmati-Abkenar M, Turner S *et al.* Weakened resilience of benthic microbial communities in the face of climate change. *ISME Commun*. 2022;**52**:21 https://doi.org/10.1038/s43705-022-00104-9
9. Li S. A warming Baltic Sea coast: Shifts in sediment microbial communities in the face of climate change. Doctoral Dissertation, Department of Biology and Encironmental Science, Linnaeus University Press, 2025
10. Li S, Salis R, Svendsen IK, Chang C, Seidel L, Sunde J *et al.* Translocation of Baltic Sea sediments impacted by 50 years of warming to present day conditions resulted in an incomplete return of microbial communities. *Unpublished manuscript*. 2025
11. Herlemann DPR, Labrenz M, Jürgens K, Bertilsson S, Waniek JJ, Andersson AF. Transitions in bacterial communities along the 2000 km salinity gradient of the Baltic Sea. *ISME J*. 2011;**5**:1571-79 https://doi.org/10.1038/ismej.2011.41
12. Hugerth LW, Wefer HA, Lundin S, Jakobsson HE, Lindberg M, Rodin S *et al.* DegePrime, a program for degenerate primer design for broad-taxonomic-range PCR in microbial ecology studies. *Appl Environ Microbiol*. 2014;**80**:5116-23 https://doi.org/10.1128/aem.01403-14
13. Stoeck T, Bass D, Nebel M, Christen R, Jones MD, Breiner HW *et al.* Multiple marker parallel tag environmental DNA sequencing reveals a highly complex eukaryotic community in marine anoxic water. *Mol Ecol*. 2010;**19**:21-31 https://doi.org/10.1111/j.1365-294X.2009.04480.x
14. Piredda R, Tomasino MP, D'erchia A, Manzari C, Pesole G, Montresor M *et al.* Diversity and temporal patterns of planktonic protist assemblages at a Mediterranean long term ecological research site. *FEMS Microb Ecol*. 2017;**93**:fiw200 https://doi.org/10.1093/femsec/fiw200
15. Straub D, Blackwell N, Langarica-Fuentes A, Peltzer A, Nahnsen S, Kleindienst S. Interpretations of environmental microbial community studies are biased by the selected 16S rRNA (gene) amplicon sequencing pipeline. *Front Microbiol*. 2020;**11**:18 https://doi.org/10.3389/fmicb.2020.550420
16. Callahan BJ, McMurdie PJ, Rosen MJ, Han AW, Johnson AJA, Holmes SP. DADA2: High-resolution sample inference from Illumina amplicon data. *Nat Methods*. 2016;**13**:581-83 https://doi.org/10.1038/nmeth.3869

17. Guillou L, Bachar D, Audic S, Bass D, Berney C, Bittner L *et al.* The Protist Ribosomal Reference database (PR2): a catalog of unicellular eukaryote small sub-unit rRNA sequences with curated taxonomy. *Nucl Acid Res*. 2012;**41**:D597-D604 https://doi.org/10.1093/nar/gks1160
18. Mitra A, Caron DA, Faure E, Flynn KJ, Leles SG, Hansen PJ *et al.* The Mixoplankton Database (MDB): Diversity of photo-phago-trophic plankton in form, function, and distribution across the global ocean. *J Eukar Microbiol*. 2023;**70**:e12972 https://doi.org/10.1111/jeu.12972
19. R Core Team. R: A Language and Environment for Statistical Computing. 2024
